# Development of Fucoxanthin-Enriched Yogurt Using Nanoliposomal Carriers: A Strategy for Functional Dairy Products with Antioxidant and Erythroprotective Benefits

**DOI:** 10.3390/molecules30081854

**Published:** 2025-04-21

**Authors:** Miguel Ángel Robles-García, Carmen Lizette Del-Toro-Sánchez, Germán Limón-Vargas, Melesio Gutiérrez-Lomelí, María Guadalupe Avila-Novoa, Fridha Viridiana Villalpando-Vargas, Brenda Vega-Ruiz, Ariadna Thalía Bernal-Mercado, Rey David Iturralde-García, Abril Ivett Priscilla Gómez-Guzman, Ernesto Ramírez-Briones, Reyna Guadalupe López-Berrellez, Ricardo Iván González-Vega

**Affiliations:** 1 Department of Medical and Life Sciences, Cienega University Center (CUCIÉNEGA), University of Guadalajara, Av. Universidad 1115, Lindavista, Ocotlán 47820, JA, Mexico; miguel.robles@academicos.udg.mx (M.Á.R.-G.); german.limon@alumnos.udg.mx (G.L.-V.); melesio.gutierrez@academicos.udg.mx (M.G.-L.); maria.anovoa@academicos.udg.mx (M.G.A.-N.); 2 Department of Research and Postgraduate in Food, University of Sonora, Blvd Luis Encinas y Rosales S/N, Col. Centro, Hermosillo 83000, SO, Mexico; thalia.bernal@unison.mx (A.T.B.-M.); rey.iturralde@unison.mx (R.D.I.-G.); 3 Department of Health Sciences, University Center of the Valleys (CUVALLE), University of Guadalajara, Carr. a Guadalajara Km. 45.5, Ameca 46600, JA, Mexico; viridiana.villalpando@academicos.udg.mx (F.V.V.-V.); brenda.vega@academicos.udg.mx (B.V.-R.); abril.gomez@academicos.udg.mx (A.I.P.G.-G.); reyna.lopez2989@alumnos.udg.mx (R.G.L.-B.); 4 Department of Cellular and Molecular Biology, University Center for Biological and Agricultural Sciences (CUCBA), University of Guadalajara, Periférico Norte N° 799 Núcleo Universitario, C. Prol. Belenes, Zapopan 45100, JA, Mexico; 5 Department of Applied Ecology, University Center for Biological and Agricultural Sciences (CUCBA), University of Guadalajara, Periférico Norte N° 799 Núcleo Universitario, C. Prol. Belenes, Zapopan 45100, JA, Mexico; ernesto.ramirez@academicos.udg.mx

**Keywords:** fucoxanthin-loaded nanoliposomes, functional foods, enriched yogurt formulation

## Abstract

In pursuing functional foods that promote health, nanoliposomal carriers have been used to enhance the stability and functionality of dairy products such as yogurt, promising therapeutic benefits. This study aimed to evaluate the impact of fucoxanthin-loaded nanoliposomes in yogurt on its antioxidant, physicochemical, and rheological properties under cold storage (21 days). Fucoxanthin-loaded nanoliposomes were prepared using the ultrasonic film dispersion technique and added at concentrations of 0%, 5%, and 10% in the yogurt (Y-C, Y-FXN-5, Y-FXN-10). Homogeneous and uniform nanoliposomes (98.28 nm) were obtained, preserving their integrity and functionality and ensuring the prolonged release and bioavailability of fucoxanthin. Y-FXN-10 maintained the highest antioxidant activity according to the DPPH (52.96%), ABTS (97.97%), and FRAP (3.16 mmol ET/g) methods. This formulation exhibited enhanced erythroprotective potential, inhibiting hemolysis, photohemolysis, and heat-induced hemolysis. However, viscosity and firmness decreased, affecting the texture and appearance. Sensory properties such as the color, flavor, aftertaste, texture, and overall acceptance improved with the 10% fucoxanthin-enriched yogurt formulation. These results suggest that nanoliposomes are suitable for carrying fucoxanthin. Their incorporation into food matrices is critical to developing functional foods. Regulatory approvals and consumer perceptions regarding nanotechnology-based products must be addressed, emphasizing their safety and health benefits.

## 1. Introduction

In a world where health and well-being are priorities, the quest for functional foods with additional health benefits has gained significant scientific interest. Dairy products like yogurt have proven effective as vehicles for delivering bioactive compounds that enhance health and prevent diseases [1]. Incorporating nanoliposomes as carriers of bioactive compounds has emerged as a promising strategy to improve their stability and bioavailability in dairy products [2,3,4]. One such compound is fucoxanthin, a marine carotenoid found in brown algae with antioxidant and potential neuroprotective effects [5]. Fucoxanthin has gained attention for its ability to inhibit oxidative stress and reduce the risk of chronic and neurodegenerative diseases [4,5,6]. The brown seaweed processing industry, particularly involving Phaeophyceae species such as *Undaria pinnatifida*, *Saccharina japonica*, and *Sargassum* spp., accounts for approximately 66% of the global seaweed production volume and about 51% of the total economic value of the algal sector, according to FAO [7]. Asia dominates this market, producing over 90% of the world’s output, with China, South Korea, and Japan as leading contributors. These seaweeds are not only important sources of hydrocolloids like alginates and fucoidans, but also provide bioactive compounds such as fucoxanthin, a carotenoid of interest in the nutraceutical, cosmetic, and food industries due to its antioxidant, anti-inflammatory, and neuroprotective properties [8]. However, its application in dairy products has been limited by low water solubility and susceptibility to degradation during processing and storage. Encapsulating fucoxanthin in nanoliposomes offers a promising solution, as they are controlled-release systems that protect and stabilize bioactive compounds, enhancing their solubility and bioavailability [6].

Nanoliposomes have emerged as promising delivery vehicles for bioactive compounds like carotenoids [4,9]. The absorption of carotenoids in the gastrointestinal system is influenced by their water solubility and the presence of lipids. Studies have shown that nanoliposomes significantly improve the bioavailability and absorption of carotenoids compared to conventional formulations [10,11]. Encapsulation in nanoliposomes also protects carotenoids from oxidative degradation and enhances their stability during food processing and storage [9,12,13]. The impact of storage on the antioxidant, physicochemical, and rheological properties of yogurt enriched with antioxidant compounds is crucial in developing functional dairy products [14,15]. During extended storage, antioxidant compounds in yogurt may be affected by oxidation, reducing their antioxidant capacity [16].

The prolonged storage of enriched yogurt can change its physicochemical properties, such as its acidity, pH, viscosity, and texture. The acidification of yogurt during storage can affect its taste and acceptability [17,18]. Additionally, changes in the structure of the dairy matrix and the distribution of added compounds can affect the homogeneity and stability of the product. The rheology of yogurt, i.e., its behavior under deformation and flow, can change during storage due to the reorganization of the dairy structure. This can manifest in changes in the yogurt’s viscosity, elasticity, and fluidity [19,20]. The formation of whey and phase separation can also occur during prolonged storage, affecting the product’s texture and consistency [19,20]. Therefore, it is important to conduct stability studies during storage to evaluate how these changes impact the quality and acceptability of enriched yogurt [21,22]. These studies will provide a better understanding of the mechanisms of deterioration during storage and help develop strategies to improve the stability and quality of yogurt enriched with antioxidant compounds, analyzing the impact of nanoliposome addition on the antioxidant, physicochemical, rheological, and sensory properties.

Thus, the present study aims to develop and evaluate yogurt enriched with fucoxanthin-loaded nanoliposomes, investigating the impact of storage on its antioxidant, physicochemical, and rheological properties under storage conditions. This multidisciplinary approach combines the encapsulation technology of bioactive compounds with food science and nutrition, aiming to provide a functional dairy product that can enhance the health and well-being of consumers. The results obtained will provide valuable insights into the feasibility of this fortification strategy and its implications for the formulation of functional foods in the future.

## 2. Results and Discussion

### 2.1. Morphological Study and Particle Size Measurement

**Figure 1:**
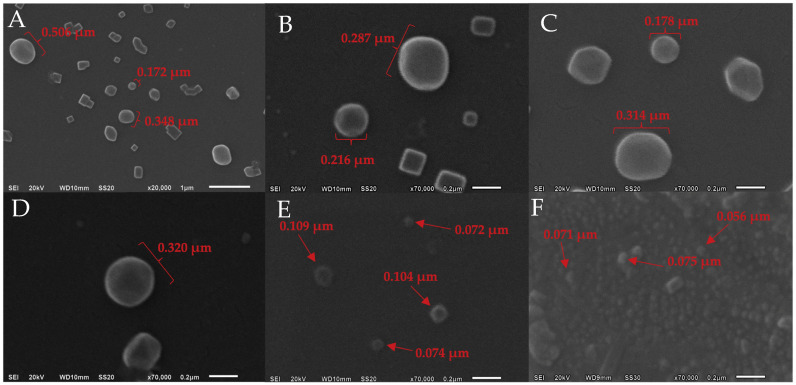
Morphology and particle size of nanoliposomes loaded with fucoxanthin by scanning electron microscopy (SEM). (**A**) Expansion to ×20,000. Scale bar = 1 µm. (**B**–**F**) Expansion to ×70,000. Scale bar = 0.2 µm.

Scanning electron microscopy (SEM) was employed to visually validate the uniformity, size, shape, and integrity of the Fucoxanthin-loaded nanoliposomes vehicles, as shown in Figure 1. For the synthesis of nanoliposomes, it was necessary to adjust key parameters such as the amplitude, duration, and type of ultrasound used to ensure vesicle stability and reduce their size to the nanometric scale. Initially, an ultrasound bath was employed, yielding liposomes with sizes that were not considered optimal for their intended application. Subsequently, high-energy ultrasound methods were tested, adjusting the amplitude to achieve an effective reduction in the particle size. The images obtained through SEM illustrate the variation in nanoliposome sizes depending on the synthesis method used. In Figure 1A,B, nanoliposomes obtained via an ultrasound bath exhibited larger sizes (0.506 µm, 0.348 µm, and 0.287 µm), indicating that this method is not efficient for significantly reducing the particle size. In contrast, Figure 1C,D, where high-energy ultrasound with a 30% amplitude was applied, show a considerable decrease in nanoliposome size (0.320 µm, 0.216 µm, and 0.178 µm), resulting in a more homogeneous and stable structure compared to the ultrasound bath. Finally, in Figure 1E,F, increasing the ultrasound amplitude to 55% led to a further reduction in the nanoliposome size (0.109 µm, 0.075 µm, and 0.056 µm). At this stage, the acoustic cavitation generated by bubbles allowed for a more efficient reduction in vesicle size, achieving a much more homogeneous particle distribution within the nanometric range.

Once the optimal conditions for obtaining nanoliposomes are determined (Figure 1E,F), the shape of the lipid vesicles confirms that all particles exhibit a similar size and structure, which are characteristic of ideal nanoliposomes [23]. Moreover, no deformations, aggregations, or structural aberrations are observed in the nanoliposomes. In research, developing new delivery systems for bioactive compounds in food matrices is a highly attractive strategy for enhancing the biological functions of these molecules. The nanoscale size of liposomes facilitates their internalization into cells and biological tissues, improving aspects such as bioavailability, especially in complex membranes like the blood–brain barrier. Their well-defined spherical shape provides greater structural support and stability, allowing the uniform distribution of encapsulated compounds within various matrices. The absence of deformations in the vesicles ensures the stability of the encapsulated compounds during storage [24,25,26].

### 2.2. Encapsulation Efficiency

The encapsulation efficiency (EE) is a measure that indicates the percentage of fucoxanthin successfully encapsulated within the liposomal vehicles relative to the total amount of fucoxanthin used in the encapsulation process (free fucoxanthin). EE is a key indicator for estimating the stability of the liposomal system, which suggests that fucoxanthin is well protected within the liposomes and is crucial for maintaining its stability during storage and administration. The encapsulation efficiency of fucoxanthin in nanoliposomes (95.33 ± 1.34%) is considered high and efficient, indicating that the encapsulation process used in this study is highly effective. The results suggest that the process parameters, such as the lipid ratio, encapsulation technique, and operating conditions, have been adequately controlled to maximize the incorporation of fucoxanthin into the nanoliposomes. This high encapsulation percentage ensures fucoxanthin’s protection and stability and opens doors for future research and practical applications in various fields. The ability to consistently maintain a high EE reinforces the viability of these nanoliposomes as efficient delivery vehicles for bioactive compounds, offering a robust platform for use in commercial and therapeutic products.

Due to the nonpolar nature of fucoxanthin, its encapsulation likely occurs in the hydrophobic part of the vehicles, specifically within the lipid membrane between the hydrophobic tails of the fatty acids constituting the phospholipids. This characteristic increases the incorporation of fucoxanthin into the liposomal vehicles. A similar encapsulation efficiency has been observed in studies by Pan et al. [27], Rodríguez-Ruiz et al. [28], and Taksima et al. [29] for astaxanthin, with efficiencies ranging from 95–97%. The hydrophobic nature of carotenoids enhances their affinity for the hydrophobic part of phosphatidylcholine molecules, encapsulating them within the lipid bilayer and facilitating their interaction and encapsulation [26,27,28,29].

### 2.3. Stability of Nanoliposomes by Centrifugation

The stability of fucoxanthin-loaded nanoliposomal carriers indicates a high degree of stability (92.7 ± 2.3%), suggesting that the nanoliposomes effectively maintain their structural integrity and functional efficacy under the given conditions. Several key points emerge when comparing the stability of these nanoliposomes to other systems reported in the scientific literature. According to a study by Sun et al. [10], nanoliposomes encapsulating natural antioxidants, such as resveratrol and curcumin, demonstrated stability percentages ranging from 85% to 90% over one month at room temperature. The observed stability of 92.7% for fucoxanthin-loaded nanoliposomes is slightly higher, indicating the better preservation of encapsulated compounds under similar conditions. Research by Mozafari et al. [30] on nanoliposomes encapsulating bioactive compounds reported stability values of approximately 88% over a similar timeframe. The higher stability percentage in the current study suggests that the formulation and preparation method for fucoxanthin-loaded nanoliposomes may be more effective in maintaining the structural integrity than the methods used in the referenced studies. The lipid composition of nanoliposomes plays a crucial role in their stability. Studies by Mozafari [31] highlight that including cholesterol in the lipid bilayer can significantly enhance the nanoliposome stability. The 92.7% stability observed in the current study might be attributed to an optimized lipid composition, possibly including cholesterol or other stabilizing agents. Storage conditions also influence the stability of nanoliposomes. Wang et al.’s [32] study on nanoliposomes containing polyphenols reported stability percentages of around 87% when stored at 4 °C. The stability of fucoxanthin-loaded nanoliposomes at 92.7% could imply that the storage conditions were optimized, possibly involving controlled temperature and light exposure to minimize degradation. This stability for fucoxanthin-loaded nanoliposomes is noteworthy and compares favorably with other nano-liposomal systems reported in the literature.

The stability of nanoliposomes is crucial for their incorporation into functional foods, such as enriched yogurt. High stability ensures that nanoliposomes maintain their structure during the processing and storage of yogurt, preserving their encapsulating properties and preventing the premature release of bioactive compounds. Stability contributes to the homogeneous distribution of bioactive compounds within the yogurt, guaranteeing a uniform concentration of the active ingredient in each serving, thus enhancing the consistency and efficacy of the functional product [33,34]. Stable nanoliposomes minimize undesirable changes in the sensory properties of yogurt, such as the flavor, texture, and color, as the degradation of unstable nanoliposomes could release compounds that negatively affect the product’s acceptability to consumers [35]. Furthermore, the stability of nanoliposomes influences the preservation of the biological activity by providing a protective barrier for bioactive compounds, shielding them from adverse factors such as light, oxygen, and temperature, thereby preserving their biological activity throughout the shelf life of products. This stability also allows for the controlled and sustained release of bioactive compounds, improving their bioavailability and efficacy, ensuring that health benefits are maintained over time [36,37]

Nanoliposome stability ensures that bioactive compounds reach their biological target in their most effective form, optimizing their antioxidant and anti-inflammatory effects in the organism. When compared to the delivery of other antioxidant compounds, resveratrol-loaded nanoliposomes exhibited a stability of 90% and significantly improved the bioavailability of resveratrol in functional foods, suggesting that fucoxanthin-loaded nanoliposomes with a stability of 92.7% could have a similar or superior impact [30,31]. Curcumin-loaded nanoliposomes, with a stability of 88%, were successfully utilized in enriched dairy products, preserving their bioactive properties, indicating that the higher stability of fucoxanthin-loaded nanoliposomes could better conserve their health benefits when incorporated into yogurt [10]. Polyphenol-loaded nanoliposomes, with a stability of 87%, maintained their antioxidant activity in functional foods, suggesting that the high stability of fucoxanthin-loaded nanoliposomes will not only preserve their biological activity, but also potentially enhance the antioxidant benefits of enriched yogurt [38]. These studies indicate that the high stability of nanoliposomes is a determinant factor for their successful incorporation into enriched yogurt, ensuring the protection and controlled release of bioactive compounds, preserving their biological activity, and enhancing the functional properties of yogurt, indicating a promising application in the functional food industry.

### 2.4. In Vitro Release

**Figure 2:**
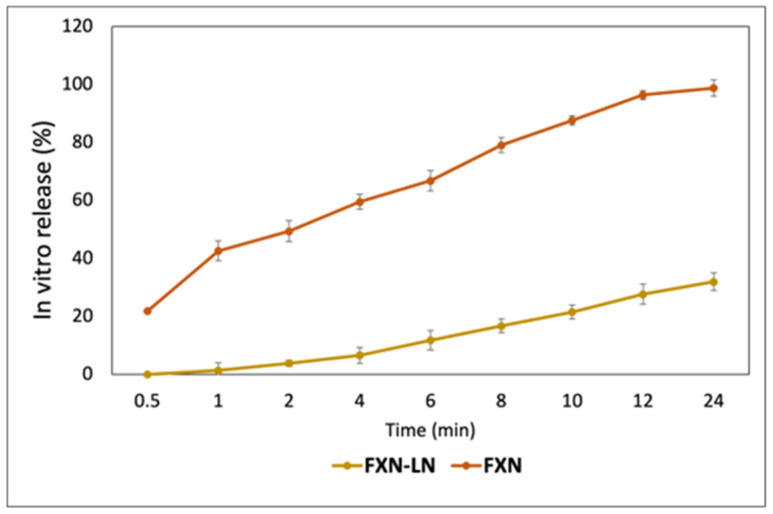
In vitro release of free fucoxanthin solution (FXN) and fucoxanthin-loaded nanoliposomes (FXN-LN) in PBS (pH 7.4) at 37 °C. Data are presented as mean ± standard deviation (*n* = 3).

As shown in Figure 2, the in vitro release profile results demonstrate that free fucoxanthin exhibits a rapid and significant release, reaching 98.8% within 24 h, with an initial increase of 21.9% at 0.5 h. This percentage quickly rises, reaching 42.6% at 1 h and 66.9% at 6 h. By 10 h, the release reaches 87.6%, and finally, at 24 h, an almost complete release is observed. In contrast, fucoxanthin encapsulated in nanoliposomes shows a slower and more controlled release, achieving only 31.9% at the end of the same period, with no release in the first 0.5 h. The release gradually increases, reaching 1.4% at 1 h and 6.6% at 4 h. At 6 h, the release is 11.8%, and it continues to increase at a constant rate, reaching 21.5% at 10 h. This difference indicates that nanoliposomes effectively delay and maintain a sustained release of fucoxanthin, which could be beneficial in applications requiring a prolonged compound release, such as drug delivery or nutritional supplements. Nanoliposome encapsulation provides a controlled and gradual release, whereas the free form offers the immediate and rapid availability of fucoxanthin. In other words, the release of free fucoxanthin is much faster, with a high percentage of release achieved in the initial hours, indicating immediate and rapid availability in its free form. Meanwhile, the release of fucoxanthin encapsulated in nanoliposomes is significantly slower and controlled. Both forms of fucoxanthin show a continuous increase in the release percentage over time, but the magnitude and rate of increase differ significantly. To describe the mathematical expression of the in vitro release of fucoxanthin (FXN) and fucoxanthin encapsulated in nanometric liposomes (FXN-LN) based on the provided figure, we can fit the data to a linear release equation. The typical linear release equation can be expressed as:

(1) $$Release\;\left(\%\right)=kt+C$$

where *k* is the release constant (slope of the line), *t* is the time, and *C* is the y-intercept (the initial release value at time zero).

The effectiveness of nanoliposomes in delaying and maintaining a sustained release of fucoxanthin has been well-documented in the scientific literature. These characteristics benefits applications requiring prolonged compound release, such as drug delivery or nutritional supplements. Unlike free fucoxanthin, which offers immediate and rapid availability, nanoliposomes enable a controlled and gradual release. Studies have shown that nanoliposome-based formulations can increase stability, improve epithelial permeability and bioavailability, and prolong the drug’s half-life in the bloodstream, while minimizing adverse side effects [39,40,41]. Additionally, nanoliposomes’ ability to encapsulate hydrophobic and hydrophilic drugs and release them in controlled amounts has been highlighted as a significant advantage in various biomedical and food applications [42,43]. These systems have shown efficacy in enhancing drug stability and improving targeted drug delivery, crucial in treating complex diseases such as cancer [44].

### 2.5. Chemical Characterization

The results presented in Table 1 demonstrate that the incorporation of fucoxanthin-loaded nanoliposomes into yogurt slightly influenced its proximate composition. The results of the proximate analysis indicate that the incorporation of fucoxanthin-loaded nanoliposomes into yogurt did not result in statistically significant differences in the dry matter or moisture content among the evaluated samples. However, a gradual increase in the dry matter content was observed with higher concentrations of nanoliposomes: the control yogurt (Y-C) showed 13.5%, while Y-FXN-5 and Y-FXN-10 reached 14.2% and 14.6%, respectively. Correspondingly, the moisture content slightly decreased, with values of 86.5% for Y-C, 85.8% for Y-FXN-5, and 85.3% for Y-FXN-10. These changes may be attributed to the lipid and structural contribution of the nanoliposomes, which promote a slight increase in the solid fraction of the product. From a technological perspective, this modification may enhance the yogurt’s texture and consistency. The slight reduction in moisture supports the greater stability during storage. Additionally, nanoliposomes may improve the bioavailability of the incorporated fucoxanthin [45].

The protein content remained relatively stable across all formulations, ranging from 37.3% in the control yogurt (Y-C) to 35.9% in the yogurt enriched with 10% nanoliposomes (Y-FXN-10), with no statistically significant differences. This suggests that the addition of nanoliposomes did not substantially affect the protein concentration, maintaining the nutritional quality of the yogurt in terms of protein. In comparison, the fat content showed a gradual increase with higher levels of nanoliposome enrichment. The control yogurt (Y-C) had 31.6% fat, which rose to 33.5% and 34.1% in Y-FXN-5 and Y-FXN-10, respectively. This increase is consistent with the lipid-based nature of the nanoliposomes used for fucoxanthin encapsulation. Although the differences in the fat content were not statistically significant, the trend suggests that nanoliposome addition contributes to a higher lipid proportion, which could enhance the yogurt’s energy content and possibly its texture and mouthfeel.

The carbohydrate and ash contents also reflected the influence of nanoliposome enrichment. A decreasing trend was observed in the carbohydrate content from 24.9% in Y-C to 22.2% in Y-FXN-10, likely due to the relative dilution effect caused by the increase in fat and ash components. Conversely, the ash content increased progressively from 6.5% in the control to 7.9% in the 10% enriched yogurt, possibly indicating the presence of minerals associated with the nanoliposome formulation. Overall, these findings suggest that while nanoliposome incorporation does not significantly alter the protein levels, it modestly affects the fat, carbohydrate, and ash content, contributing to a slightly richer and potentially more potentially functional yogurt matrix.

Fucoxanthin-loaded nanoliposome-enriched yogurts exhibit a distinct nutritional composition compared to other types of yogurts reported in the scientific literature. The primary difference lies in the higher fat content, which is attributable to the incorporation of lipid-based nanoliposomes. These variations in the proximate composition may influence the organoleptic properties and nutritional profile of the final product, suggesting that the addition of fucoxanthin-loaded nanoliposomes may offer specific benefits that differ from other types of yogurt fortification [45].

### 2.6. Microbiological Quality of Yogurt

The microbiological analysis of artisanal yogurt enriched with fucoxanthin-loaded nanoliposomes demonstrated that all formulations, both control and enriched (5% and 10%), complied with the permissible limits established by the NOM-243-SSA1-2010 [46] for fermented dairy products. The total mesophilic aerobic counts in all samples exceeded the minimum threshold of 1.0 × 10^6^ CFU/g, which is indicative of a healthy population of lactic acid bacteria, as expected in fermented products [34]. Moreover, the results of the control yogurt (Y-C) showed a count of 1.2 × 10^7^ CFU/g, while the yogurt enriched with 5% fucoxanthin-loaded nanoliposomes (Y-FXN-5) exhibited 9.4 × 10^6^ CFU/g, and the yogurt with 10% nanoliposomes (Y-FXN-10) showed a slightly lower value of 8.7 × 10^6^ CFU/g. These viable populations are essential for maintaining the characteristic fermentation, flavor, and probiotic potential of yogurt [41].

The absence of total and fecal coliforms in all samples indicates excellent hygienic conditions during yogurt production and handling, aligning with the sanitary requirements defined by the NOM-243-SSA1-2010 [46]. This absence is particularly relevant given the artisanal nature of the yogurt and confirms the effectiveness of standardized procedures and fermentation control implemented during preparation. Moreover, the complete absence of molds and yeasts in all samples, including those enriched with fucoxanthin, suggests that the storage and formulation conditions effectively inhibited fungal growth. The lack of fungal contamination in enriched samples could also be associated with the moderate antimicrobial and antifungal properties of fucoxanthin, previously reported in the scientific literature, although this was not the primary focus of this study. These results collectively confirm that the incorporation of nanoliposomes loaded with fucoxanthin does not compromise the microbiological quality or safety of artisanal yogurt and maintains the microbial profile required for probiotic and sensory functionality, as described in previous studies [34,35].

Subtle differences were observed in the microbial counts compared to the yogurt without nanoliposomes. These variations were likely attributable to the antioxidant and mildly antimicrobial activity of the encapsulated fucoxanthin, without compromising the viability of the lactic acid starter cultures [5]. Therefore, all yogurt formulations complied with the microbiological limits established by the NOM-243-SSA1-2010, indicating that the products were safe for human consumption. These results confirmed the hygienic quality of the yogurt samples, thereby ensuring their suitability for a subsequent sensory evaluation.

### 2.7. Impact of Cold Storage Conditions on Antioxidant, Physicochemical, and Rheological Properties of Different Yogurt Formulations

#### 2.7.1. Antioxidant Properties

Cold storage is a common practice to prolong the shelf life of food and preserve its functional and sensory properties. This study evaluated the antioxidant properties of yogurt formulations (Y-C, Y-FXN-5, Y-FXN-10) under a 21-day cold storage period (Table 2). The DPPH, ABTS, and FRAP methods assessed the antioxidant properties. The DPPH and ABTS methods measure a substance’s antioxidant capacity to donate electrons or hydrogen atoms and neutralize free radicals.

The results show a decrease in antioxidant activity for the different formulations as the storage period progresses. The free-radical scavenging activity was evaluated using the DPPH assay: For Y-C, the antioxidant activity decreased from 24.77% to 19.23% by day 21. Initially, Y-FXN-5 exhibited an antioxidant activity of 34.99%, which decreased to 31.06% at the end of the storage period. Finally, Y-FXN-10 showed a much higher initial antioxidant activity (52.9%), which was reduced to 50.3% by day 21. Thus, the results indicate that the Y-FXN-10 formulation maintained the highest DPPH free-radical inhibitory activity. The ABTS assay also measures the antioxidant capacity but, unlike DPPH, can react with a broader range of antioxidant molecules. The results show that the antioxidant activity of the control (Y-C) decreased from 33.7% to 25.9% after 21 days. In Y-FXN-5, the initial antioxidant activity was 43.38%, decreasing to 39.27% at the end of the storage period. Y-FXN-10 started with a high value of 97.9% and ended at 88.4%. Regarding the reducing power of the formulations measured by FRAP, the control (Y-C) showed a decrease from 2.9 mmol ET/g to 2.3 mmol ET/g, while Y-FXN-5 showed an initial capacity of 3.2 mmol ET/g, slightly reducing to 3.2 mmol ET/g. The Y-FXN-10 formulation presented the highest initial capacity (3.3 mmol ET/g), slightly decreasing to 3.0 mmol ET/g by day 21. These results indicate that the addition of fucoxanthin-loaded nanoliposomes (Y-FXN-5 and Y-FXN-10) significantly enhances the antioxidant properties of yogurt compared to the control (Y-C), with Y-FXN-10 showing the greatest antioxidant stability during cold storage. It is observed that the nanoliposomes might be providing additional protection, possibly due to the controlled release of fucoxanthin and protection against oxidation.

Yogurt formulations without antioxidant additives or functional ingredients such as nanoliposomes typically show a decrease in their antioxidant capacity during storage [47] This is due to the natural degradation of antioxidant compounds like vitamins, polyphenols, and bioactive peptides. Studies by Corrêa et al. [48] report that the antioxidant capacity in unenriched yogurts significantly decreases during storage, similar to what was observed in the Y-C formulation of our study. Bourne [49] and Lee and Lucey [50] reported a decrease in the antioxidant capacity in natural yogurts due to the oxidation of lipids and proteins during cold storage. In our study, the Y-C formulation showed a significant reduction in the antioxidant capacity (DPPH, ABTS, FRAP) over the 21-day storage period, consistent with Lee and Lucey’s [50] findings.

On the other hand, natural antioxidants have been incorporated into yogurts to enhance their functional properties. According to studies by Jeong et al. [51], yogurt enriched with green tea extract significantly improved the initial antioxidant capacity, but decreased during storage. Zahoor et al. [52] reported that adding polyphenol-rich fruit extracts derived from pomegranate and açaí increased the initial antioxidant capacity of yogurt. However, the antioxidant capacity decreased in both studies over time due to the degradation of bioactive compounds that were not protected or encapsulated. Using nanoliposomal carriers to transport bioactive compounds and effectively achieve a controlled release of antioxidants is a relatively new and promising strategy. Studies by Gómez-Estaca et al. [53] found that curcumin-loaded nanoliposomes significantly improved yogurt’s stability and antioxidant capacity during storage. Encapsulation showed a lesser decrease in the antioxidant capacity compared to non-encapsulated yogurts. Similarly, Kristl et al. [54] found that resveratrol-loaded nanoliposomes not only improved the initial antioxidant capacity of yogurt, but also maintained these properties during a prolonged cold storage period. The results of both studies are consistent with our findings aimed at improving the stability of antioxidants in yogurt. Based on previous studies and the results obtained in our research, incorporating nanoliposomes in yogurts can be an effective strategy to develop functional dairy products such as yogurt with enhanced antioxidant benefits, potentially contributing to consumer health and extending the shelf life of products. These findings suggest that incorporating fucoxanthin-loaded nanoliposomes in yogurt improves its initial antioxidant properties and helps maintain these properties during cold storage, making the product more stable and potentially healthier for consumers. However, these results are insufficient to establish the therapeutic potential of yogurt formulations to delay cellular oxidation and premature aging. Additionally, the erythroprotective activities (hemolysis inhibition, photohemolysis inhibition, and the membrane stabilization test) will provide complementary information on the cellular antioxidant activity.

Compared with other studies, similar trends are observed where nanoliposome encapsulation enhances the stability and retention of antioxidant properties in food matrices. For instance, quercetin-loaded nanoliposomes showed sustained antioxidant effects and improved physicochemical stability, similar to the color stability observed in fucoxanthin-loaded yogurt samples. Quercetin encapsulation in nanoliposomes improved the retention of antioxidant activity and influenced the release profiles, providing a prolonged protective effect against oxidative damage [55]. Another study on rosemary oleoresin-loaded nanoliposomes demonstrated enhanced antioxidant stability and significant improvements in the oxidative stability. The rosemary oleoresin nanoliposomes maintained high antioxidant activity over an extended storage period, paralleling the sustained color stability in the fucoxanthin-enriched yogurt samples. This reinforces the efficacy of nanoliposome encapsulation in preserving the functional properties of antioxidants during storage [12]. These comparisons indicate that nanoliposome technology consistently enhances the stability and effectiveness of encapsulated antioxidants across different food systems.

Table 3 shows the results of erythroprotective activities. Hemolysis inhibition (HI%) was significantly higher in the yogurt formulations enriched with nanoliposomes (Y-FXN-5 and Y-FXN-10) compared to the control yogurt (Y-C). Y-FXN-10 exhibited the highest hemolysis inhibition at 82.4%, followed by Y-FXN-5 at 63.8%, whereas the control only reached 17.3%. These data are consistent with the results for the percentage of photohemolysis inhibition, another method of inducing free-radical production. Y-FXN-10 also showed a notably higher erythroprotective potential at 82.4%, significantly higher than the control (61.0%) and Y-FXN-5 (54.9%). Regarding the membrane stabilization test, for Heat-IH, Y-FXN-10 presented significantly higher values (46.8%) compared to Y-C (25.5%) and Y-FXN-5 (25.2%). Although the difference is not as marked as in the other parameters, these results indicate that incorporating nanoliposomal vehicles at a 10% concentration contributes to the stability of erythrocytes under thermal stress conditions. While stabilizing the membrane by Hypo-IH, the Y-FXN-10 formulation again exhibited the highest inhibition (93.6%), followed by Y-C (81.5%) and Y-FXN-5 (80.9%).

The results clearly demonstrate that adding FXN-LN significantly enhances the erythroprotective potential of the yogurt formulations. Hemolysis inhibition, photohemolysis inhibition, and the membrane stabilization test (heat-induced hemolysis inhibition, hypotonicity-induced hemolysis inhibition) were the parameters that showed the most notable differences between the enriched formulations and the control, indicating that the antioxidant compounds present in the enriched yogurt have a protective effect against oxidative damage. This can be attributed to fucoxanthin being a potent antioxidant, and its encapsulation in nanoliposomes can improve its stability and bioavailability. Therefore, the incorporation of nanoliposomes loaded with fucoxanthin not only enhances the antioxidant capacity, stability, and bioavailability of fucoxanthin in yogurt formulations, but also potentiates its protective effect under various stress conditions, such as light and heat exposure, which are common in the food industry. These nanoscale vesicles could also confer an antioxidant power comparable to a food preservative, prevent lipid peroxidation, and extend the shelf life of products [47].

**Figure 3:**
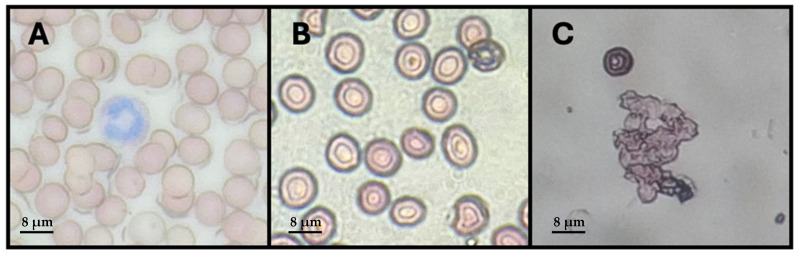
Evaluation of the erythroprotective potential of a yogurt formulation enriched with fucoxanthin-loaded nanoliposomes in human erythrocytes exposed to oxidative stress. (**A**) Healthy erythrocytes (negative control). (**B**) Erythrocytes treated with a yogurt formulation enriched with fucoxanthin-loaded nanoliposomes, indicating a protective effect against oxidative damage. (**C**) Erythrocytes exposed to AAPH, a free-radical generator.

Figure 3 presents three micrographs (A, B, and C) illustrating the morphological state of human erythrocytes under different experimental conditions. In Figure 3A, healthy erythrocytes exhibit their characteristic morphology: biconcave cells with a round and uniform shape, homogeneous coloration, and no apparent alterations in the plasma membrane. The biconcave structure is essential for the erythrocyte function, as it maximizes the surface area for gas exchange and ensures optimal deformability for passage through the capillaries. Additionally, a bluish-stained cell, likely a leukocyte, can be observed, which is a common finding in blood smears. Under these basal conditions, erythrocytes maintain their structural integrity without signs of hemolysis or deformation, indicating a physiologically stable environment free from significant oxidative stress.

In Figure 3B, erythrocytes were exposed to a yogurt formulation enriched with fucoxanthin-loaded nanoliposomes, an antioxidant with protective activity against oxidative damage. Most erythrocytes retain their characteristic biconcave morphology, although slight membrane alterations are observed, possibly due to interactions with the antioxidant. The cellular structure remains largely uniform, with fewer altered cells compared to Figure 3C. In contrast, Figure 3C shows erythrocytes exposed to AAPH, a free-radical generator that induces oxidative stress. Here, a significant alteration in the cellular morphology is evident, with fragmented, hemolyzed erythrocytes and a loss of their biconcave structure. The plasma membrane appears visibly damaged, suggesting severe lipid peroxidation that compromises the cell viability. Comparatively, Figure 3B confirms the erythroprotective effect of fucoxanthin-enriched yogurt, as it largely prevents the oxidative damage observed in Figure 3C, highlighting the critical role of antioxidants in cellular protection.

#### 2.7.2. Physicochemical Properties

##### Electric Conductivity

**Figure 4:**
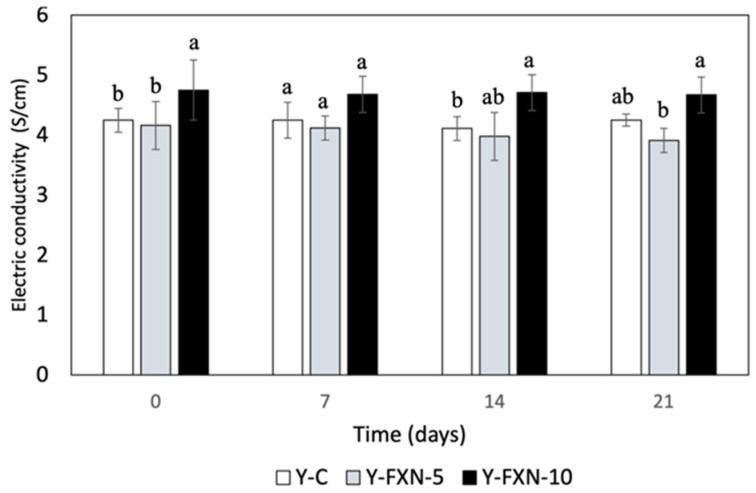
Effect of fucoxanthin-loaded nanoliposome addition and cold storage time on the electrical conductivity of yogurt formulations. All data were analyzed using two-way ANOVA with interactions. Means with different superscript (a,b: pH effects) within the same treatment are significantly different. Y-C = Control Yogurt without nanocapsules; Y-Ant-5 = Yogurt enriched with 5% nanocapsules; Y-Ant-10 = Yogurt enriched with 10% nanocapsules. Bars represent the standard deviation of at least three replicates (*n* > 3) per concentration.

The electrical conductivity of yogurt formulations was affected by the incorporation of fucoxanthin-loaded nanoliposomes and cold storage over 21 days (Figure 4). The control yogurt (Y-C) maintained stable conductivity (~4.3 S/cm), while yogurt with 5% fucoxanthin (Y-FXN-5) showed a greater decline (4.16 to 3.91 S/cm), indicating a possible ionic imbalance or matrix instability. In contrast, yogurt with 10% fucoxanthin (Y-FXN-10) exhibited a minor reduction (4.7 to 4.6 S/cm), suggesting a stabilizing effect at higher concentrations. Electrical conductivity, a key indicator of matrix homogeneity and product quality, reflects modifications in the ionic balance due to nanoliposome dispersion [56]. Significant variations could indicate undesirable interactions affecting the texture and flavor. These findings align with previous studies on bioactive nanocarriers in dairy products, where the nanoliposome encapsulation of antioxidants, such as curcumin, influenced the electrical properties [57,58,59]. The observed conductivity changes reinforce the relevance of nanotechnology in enhancing yogurt stability and functionality.

##### pH

**Figure 5:**
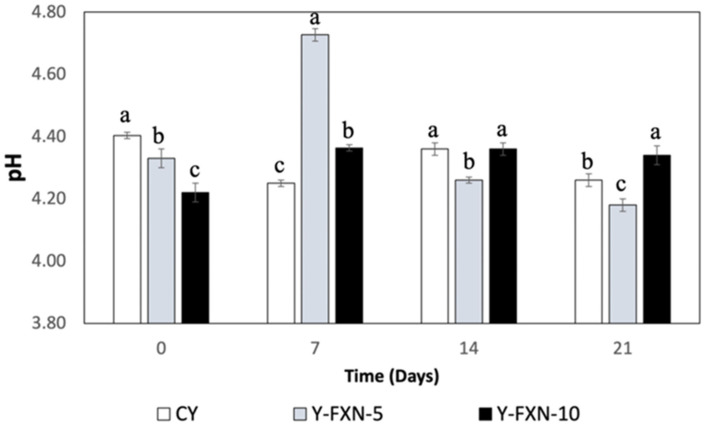
Effect of fucoxanthin-loaded nanoliposome addition and cold storage time on the pH of yogurt formulations. All data were analyzed using two-way ANOVA with interactions. Means with different superscript (a–c: pH effects) within the same treatment are significantly different. Y-C = Control Yogurt without nanocapsules; Y-FXN-5 = Yogurt enriched with 5% nanocapsules; Y-FXN-10 = Yogurt enriched with 10% nanocapsules. Bars represent the standard deviation of at least three replicates (*n* > 3) per concentration.

The analysis of pH changes in yogurt formulations over 21 days of cold storage indicates that fucoxanthin-loaded nanoliposomes influence acidification dynamics (Figure 5). The control yogurt (Y-C) exhibited a slight pH decline (4.4 to 4.3) due to the lactic cultures’ metabolic activity. Yogurt with 5% fucoxanthin nanoliposomes (Y-FXN-LN-5%) showed a more pronounced pH decrease (4.3 to 4.2), suggesting increased acidogenic activity or acid release from nanoliposomes. In contrast, yogurt with 10% fucoxanthin nanoliposomes (Y-FXN-LN-10%) exhibited a milder pH change (4.2 to 4.3), indicating potential stabilization effects, possibly through buffering or interference with lactic culture activity. These findings align with prior research showing that nanoencapsulation enhances carotenoid stability, bioaccessibility, and physicochemical properties, including pH regulation [60,61,62]. Studies on fucoxanthin- and chitosan-based delivery systems confirm their role in maintaining carotenoid integrity and modulating acidification in food matrices, reinforcing the stabilizing impact of nanoliposomes in dairy products [63].

##### Titratable Acidity

**Figure 6:**
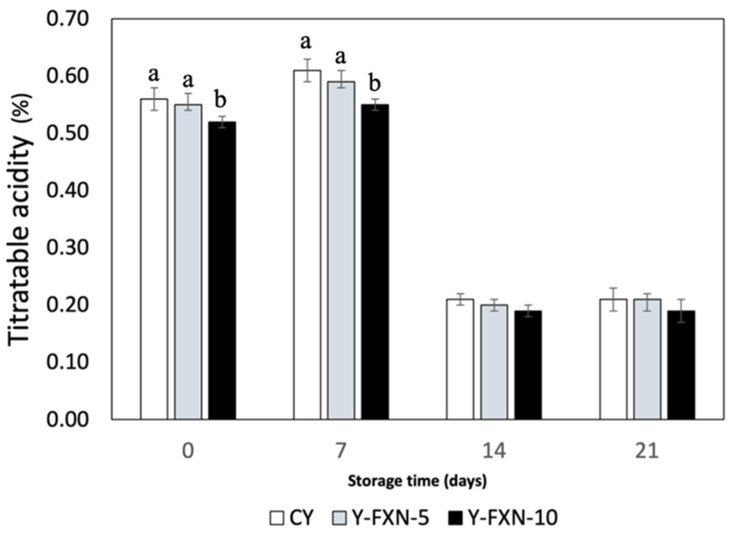
Effect of nanoliposome addition and cold storage time on the titratable acidity of yogurt formulations. All data were analyzed using one-way ANOVA with interactions. Means with different superscript (a,b: pH effects) within the same treatment are significantly different. Bars without lowercase letters indicate no significant differences. Y-C = Control Yogurt without nanocapsules; Y-FXN-5 = Yogurt enriched with 5% nanocapsules; Y-FXN-10 = Yogurt enriched with 10% nanocapsules. Bars represent the standard deviation of at least three replicates (*n* > 3) per concentration.

The titratable acidity of yogurt formulations over 21 days of cold storage showed a slight initial reduction with the addition of fucoxanthin-loaded nanoliposomes, but remained stable in the long term (Figure 6). At day 0, the control yogurt (Y-C) had an acidity of 0.56%, while formulations with 5% (Y-FXN-LN-5%) and 10% (Y-FXN-LN-10%) fucoxanthin nanoliposomes had slightly lower values (0.55% and 0.52%, respectively). The acidity increased by day 7 due to active fermentation, but declined significantly by day 14, stabilizing at lower levels by day 21. This suggests that nanoliposomes may slow acid production, contributing to yogurt stability [64]. These results align with previous studies, indicating that fucoxanthin incorporation alters the acidity without negatively affecting the physicochemical properties. Maintaining a lower initial acidity can lead to a milder flavor, preferred by some consumers, while nanoliposomes contribute to the structural integrity, texture, and extended shelf life, ensuring product quality and functional benefits over time [64,65,66].

##### Syneresis Susceptibility (STS)

**Figure 7:**
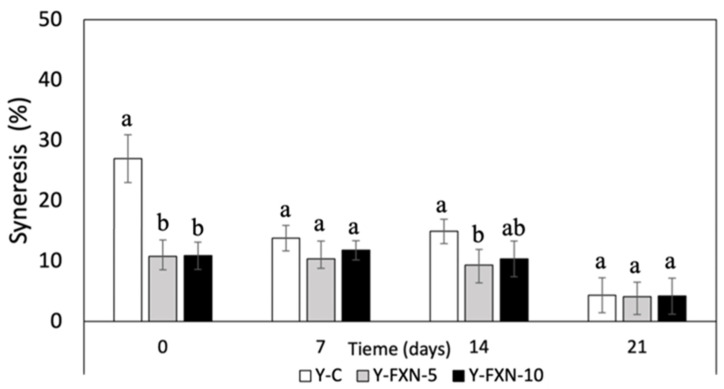
Effect of fucoxanthin-loaded nanoliposome addition and cold storage time on the syneresis susceptibility of yogurt formulations. All data were analyzed using two-way ANOVA with interactions. Means with different superscript (a,b: storage time effects) within the same treatment are significantly different. Y-C = Control Yogurt without nanocapsules; Y-FXN-5 = Yogurt enriched with 5% nanocapsules; Y-FXN-10 = Yogurt enriched with 10% nanocapsules. Bars represent the standard deviation of at least three replicates (*n* > 3) per concentration.

The addition of fucoxanthin-loaded nanoliposomes significantly reduces syneresis in yogurt formulations over 21 days of cold storage (Figure 7). The control yogurt (Y-C) initially had a syneresis value of 26.5%, which decreased substantially over time, indicating notable whey separation. Conversely, formulations with 5% and 10% nanoliposomes (Y-FXN-5 and Y-FXN-10) exhibited much lower initial syneresis values (~10.8%) and maintained stable low levels throughout storage, suggesting that nanoliposomes enhance the structural stability. This effect is attributed to nanoliposomes’ interactions with yogurt proteins, particularly casein micelles, strengthening the gel network and preventing whey expulsion [63,64]. Additionally, nanoliposomes may interact with polysaccharides and lipids, further improving the viscosity, water retention, and textural integrity. Previous studies confirm that nanoliposomes function as emulsifiers and protein stabilizers, contributing to a more cohesive and homogeneous yogurt matrix. This structural enhancement ensures an improved texture, reduced phase separation, and better overall product stability during refrigerated storage [67,68,69,70].

##### Water-Holding Capacity (WHC)

**Figure 8:**
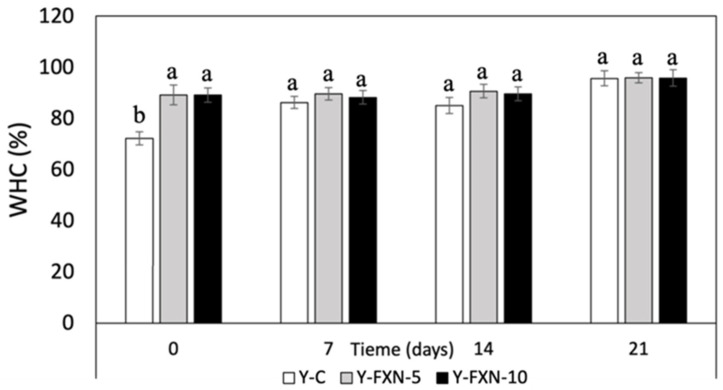
Effect of the addition of nanoliposomes and cold storage time on the water-holding capacity of yogurt formulations. All data were analyzed using two-way ANOVA with interactions. Means with different superscript (a,b: storage time effects) within the same treatment are significantly different. Y-C = Control Yogurt without nanocapsules; Y-FXN-5 = Yogurt enriched with 5% nanocapsules; Y-FXN-10 = Yogurt enriched with 10% nanocapsules. Bars represent the standard deviation of at least three replicates (*n* > 3) per concentration.

The addition of fucoxanthin-loaded nanoliposomes to yogurt formulations (Y-FXN-5 and Y-FXN-10) significantly improved the water-holding capacity over 21 days of cold storage compared to the control yogurt (Figure 8). While Y-C increased from 73.53% to 95.69%, nanoliposome-enriched formulations maintained consistently higher values from the start, with Y-FXN-5 ranging from 89.2% to 95.9% and Y-FXN-10 from 89.1% to 95.82%. This suggests that fucoxanthin enhances water retention, contributing to a better texture and stability [70,71,72]. Nanoliposomes interact with yogurt matrix components—proteins (casein and whey), carbohydrates, and lipids—creating a stable network that reduces syneresis and enhances creaminess. Their emulsifying properties further prevent phase separation, improving the overall product quality. Similar results have been observed with polymerized whey proteins and plant extracts, confirming that functional agents like nanoliposomes can enhance yogurt’s structural integrity, maintain a uniform consistency, and improve consumer acceptability [71,72,73,74].

##### Viscosity

**Figure 9:**
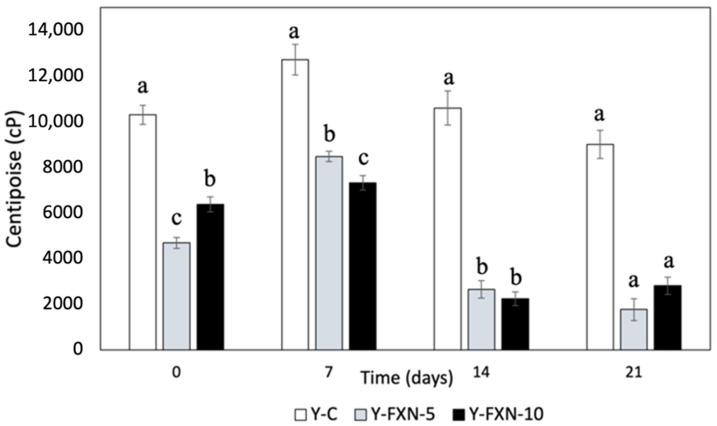
Effect of fucoxanthin-loaded nanoliposome addition and cold storage time on yogurt formulations’ viscosity (cP). Means with different superscript (a–c: storage time effects) within the same treatment are significantly different. Y-C = Control Yogurt without nanocapsules; Y-Ant-5 = Yogurt enriched with 5% nanocapsules; Y-Ant-10 = Yogurt enriched with 10% nanocapsules. Bars represent the standard deviation of at least three replicates (*n* > 3) per concentration.

The addition of fucoxanthin-loaded nanoliposomes and the cold storage duration significantly influence the viscosity of yogurt formulations (Figure 9). The control yogurt showed an initial viscosity of 10,300, peaking at 12,715 after 7 days before gradually declining to 9009 by day 21. In contrast, fortified formulations (Y-FXN-5 and Y-FXN-10) exhibited lower viscosity throughout storage, with sharp declines after 7 days, indicating the potential destabilization of the yogurt matrix. Y-FXN-5 started at 4690, peaked at 8473, and dropped to 1771 by day 21, while Y-FXN-10 followed a similar trend, peaking at 7323 before declining to 2812. These changes suggest that nanoliposomes disrupt casein micelles through electrostatic and hydrophobic interactions, reducing the micellar stability and viscosity. Additionally, nanoliposomes may act as surfactants, affecting protein aggregation, the hydration properties of polysaccharides, and fat globule interactions, ultimately altering the gel structure and rheological properties. While fortification with fucoxanthin offers nutritional benefits, its impact on viscosity and texture requires optimization to maintain the yogurt’s stability and sensory quality [71,72,73,74].

Casein micelles play a crucial role in yogurt’s gel-like texture, but their interaction with nanoliposomes can alter their size and stability. Fucoxanthin-loaded nanoliposomes may disrupt micellar structures through electrostatic and hydrophobic interactions, reducing the viscosity by weakening protein–protein interactions and promoting a more dispersed network [53]. Additionally, nanoliposomes may compete with polysaccharides for water molecules, affecting gelation and thickening properties, while their integration into fat globules can modify the size distribution and stability, influencing the yogurt’s rheology [75,76]. Studies of Shokery et al. [76], Najgebauer-Lejko et al. [77], and de Campo et al. [78] confirm that nanoliposomes can lead to reduced viscosity due to interactions with proteins and fats, impacting the stability and texture. The significant viscosity decline in fortified yogurts (Y-FXN-5 and Y-FXN-10) suggests a higher susceptibility to syneresis, as viscosity is crucial for serum retention. However, the research highlights that the water-holding capacity and gel structure stability also influence syneresis. While the control yogurt (Y-C) retained a higher viscosity and better water retention, enriched formulations exhibited structural modifications requiring optimization. Research by Anuyahong et al. [15], Liu et al. [20], and Athar et al. [79] supports the idea that increasing the total solids content and gel firmness can enhance the viscosity and reduce syneresis. These findings highlight the importance of balancing nutritional fortification with maintaining the desirable physical properties of yogurt.

##### Textural Properties (Firmness and Consistency)

**Figure 10:**
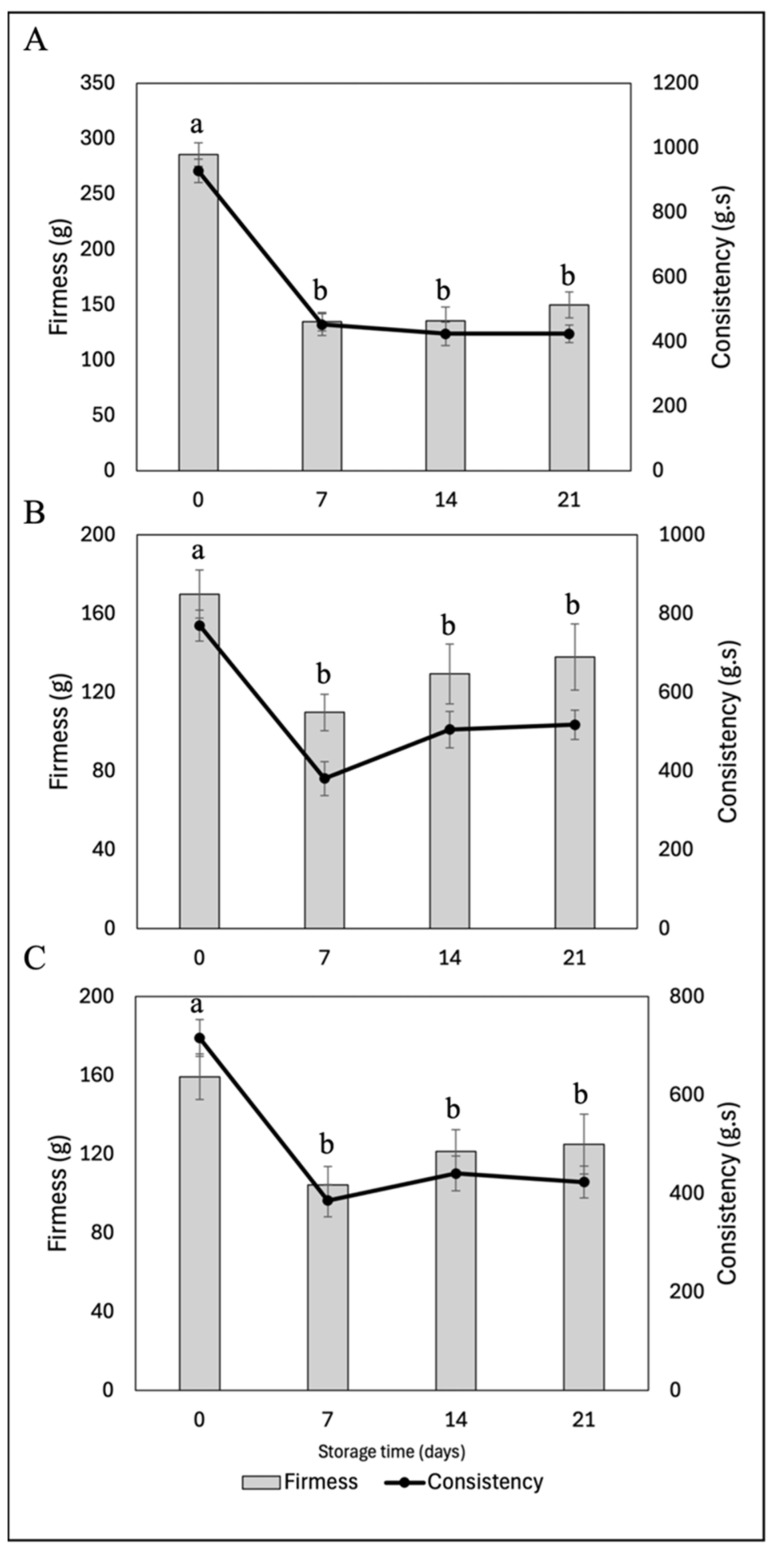
Effect of adding nanoliposome vehicles to yogurts under different cold storage times on their textural properties (firmness and consistency). (**A**) Y-C: Control Yogurt; (**B**) Y-FXN-5: Yogurt enriched with 5% of fucoxanthin-loaded nanoliposome; (**C**) Y-FXN-10: Yogurt enriched with 10% of fucoxanthin-loaded nanoliposome. Lowercase letters indicate significant differences.

The incorporation of fucoxanthin-loaded nanoliposomes affects the yogurt firmness during cold storage, depending on the concentration and interactions with the food matrix (Figure 10). The control yogurt (Y-C) exhibited an initial firmness of 285.6 g, decreasing to 149.58 g after 21 days, indicating a significant texture loss. Yogurt with 5% nanoliposomes (Y-FXN-5) showed greater stability, with firmness declining from 169.9 g to 137.9 g, suggesting a better retention of consistency. In contrast, yogurt with 10% nanoliposomes (Y-FXN-10) experienced the greatest firmness loss, dropping from 134.4 g to 54.8 g, indicating a significant structural breakdown. Firmness is a key quality parameter influencing the consumer perception, with higher firmness often associated with a better texture in spoonable yogurts. However, reduced firmness may be desirable for drinkable yogurt formulations. While Y-C maintains higher firmness, Y-FXN-5 exhibits better stability, making it a potential candidate for improving yogurt formulations without compromising texture. These findings highlight the importance of balancing nutritional fortification with sensory and structural properties to optimize consumer acceptance.

The incorporation of fucoxanthin-loaded nanoliposomes affects yogurt firmness and consistency during cold storage, with Y-FXN-10 experiencing the most significant reduction, potentially lowering consumer acceptance [67]. Nanoliposomes interact with yogurt components like caseins, proteins, lipids, and carbohydrates, influencing the protein network’s stability and modifying viscosity and texture [68,80,81]. After 21 days, Y-C showed the highest consistency retention (928.4 g·s to 424.1 g·s), while Y-FXN-5 exhibited moderate stability (769.5 g·s to 517.2 g·s), suggesting its potential for better texture maintenance, whereas Y-FXN-10 displayed the greatest consistency loss (611.5 g·s to 199.5 g·s), indicating structural breakdown. Shear stress analysis confirmed that nanoliposomes reduce the yogurt rigidity, increasing the fluidity, particularly in Y-FXN-10. These findings highlight the need to optimize the nanoliposome concentration to balance nutritional benefits with maintaining a desirable yogurt texture and stability [81,82].

##### Rheological Properties

**Figure 11:**
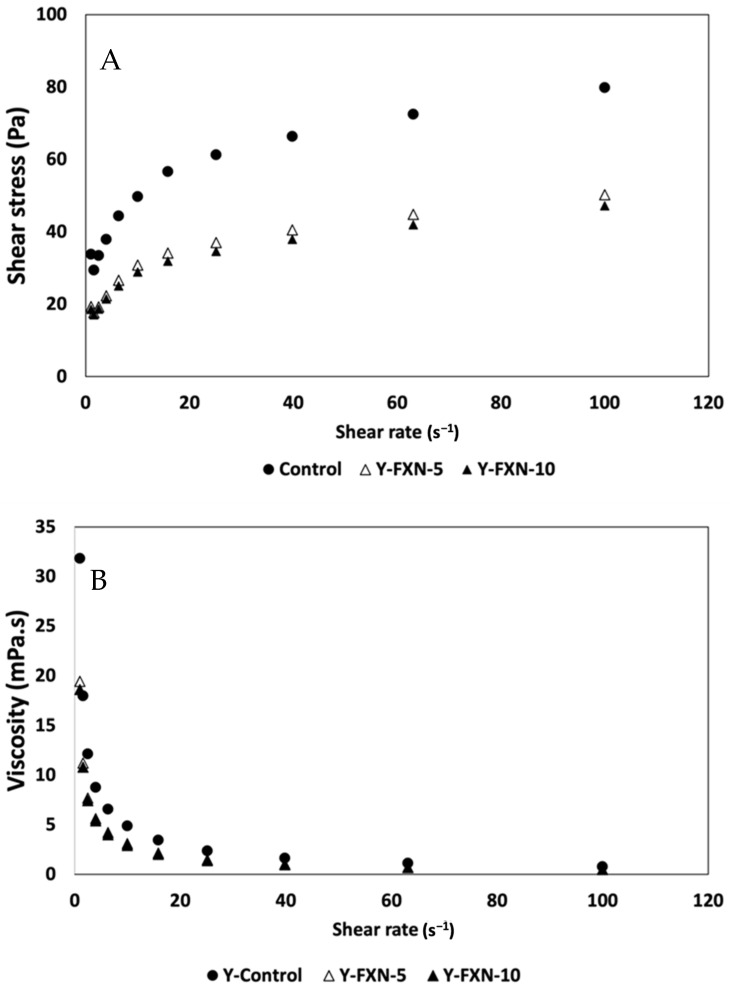
Effect of the incorporation of nanoliposomes under cold storage time on the rheological parameters: (**A**) shear stress and (**B**) viscosity of different yogurt formulations. All results were analyzed in triplicate (*n* > 3).

The addition of nanoliposomes significantly reduces the yogurt viscosity compared to the control (Y-C), likely due to interactions that alter the protein network and create a less rigid structure (Figure 11). At a shear rate of 1.00 s^−1^, Y-C has a viscosity of 31.8 mPa·s, while Y-FXN-5 and Y-FXN-10 exhibit lower values (19.4 mPa·s and 18.6 mPa·s, respectively), suggesting that nanoliposomes act as lubricants, facilitating the flow. All formulations display shear-thinning behavior, with the viscosity decreasing as the shear rate increases. At 10.00 s^−1^, Y-C maintains a viscosity of 4.9 mPa·s, whereas Y-FXN-5 and Y-FXN-10 drop to 3.09 mPa·s and 2.89 mPa·s, respectively, indicating greater susceptibility to deformation. Over 21 days, nanoliposomes contribute to maintaining a stable, less rigid structure, potentially reducing syneresis by improving water retention, which enhances the yogurt texture and consumer appeal [73].

The yogurt firmness and viscosity are key quality parameters, influencing the consumer perception of the texture and palatability. While nanoliposomes reduce the initial firmness, they enhance the creaminess and smoothness, contributing to a more desirable yogurt texture [56]. Their ability to lower shear stress may also reduce syneresis by improving water retention. Studies on the nanoencapsulation of bioactive compounds, such as fish oil, demonstrate improved stability, reduced syneresis, and enhanced sensory properties [83,84]. By interacting with casein micelles and proteins, nanoliposomes modify the gel structure, acting as emulsifiers that better distribute lipids and carbohydrates, leading to a more homogeneous texture. These findings suggest that nanoliposome integration enhances both rheological and sensory properties while preserving bioactive compound stability, reinforcing the potential of nanoencapsulation in improving the dairy product quality [85]. However, further research is needed to fully understand their behavior in food systems.

##### Sensorial Analysis

Table 4 provides a comprehensive sensory analysis detailing the evaluation conducted by a group of panelists on the sensory attributes of three different yogurt formulations (Y-C, Y-FXN-LN-5, and Y-FXN-LN-10). The attributes assessed include the color, flavor, aftertaste, aroma, consistency, texture, appearance, and overall acceptance. The sensory scale ranges from 0 (very unpleasant) to 9 (very pleasant). The incorporation of fucoxanthin-loaded nanoliposomes in the yogurt, depending on the concentration, has a positive effect on various sensory attributes. In particular, an increase in the fucoxanthin concentration (10%) improves the color, flavor, aftertaste, aroma, texture, appearance, and overall acceptance of the yogurt. Similarly, a 5% fucoxanthin concentration enhances some attributes compared to the control. However, this 5% concentration is not as effective in acceptance by the panelists as the 10% fucoxanthin concentration.

Regarding the color attribute, the yogurt enriched with 10% fucoxanthin (Y-FXN-LN-10) was the highest-rated attribute, surpassing that of Y-C. However, the yogurt with a 5% fucoxanthin concentration (Y-FXN-LN-5) received the lowest score. This indicates that a higher concentration of fucoxanthin can significantly improve the color of the yogurt, making it more visually appealing. Similarly, Y-FXN-LN-10 received the highest score for flavor, followed by the control. Y-FXN-LN-5 had the lowest score, although it remains high on the sensory scale. This suggests that higher concentrations of fucoxanthin can enhance the flavor of the yogurt. After consuming the yogurt, the aftertaste, the lingering flavor perception in the mouth, was best rated in the yogurt enriched with 10% fucoxanthin. This indicates that fucoxanthin improves the initial flavor and contributes to a more pleasant aftertaste. It is important to note that a good aftertaste is generally pleasant, complementing the initial flavor of the yogurt. Conversely, an unpleasant aftertaste can deter consumers from repurchasing the product. Regarding the aroma, consistency, texture, appearance, and overall appearance of the formulations, Y-FXN-LN-10 and Y-C were equally appreciated, while the 5% yogurt received a lower score. These results suggest that fucoxanthin is a beneficial additive for improving the sensory quality of yogurt at 10% concentrations.

According to the data provided by the sensory attribute analysis, the Y-FXN-LN-10 formulation has the highest acceptability, with a score of 8.36 on the sensory scale. This formulation also obtained the highest scores in several sensory attributes (the color, flavor, aftertaste, texture, appearance, and overall acceptance), indicating a greater preference among the panelists. The attribute “Overall Acceptance” is the most important, as this information is crucial for concluding which formulation was most accepted by the panelists. Although other attributes such as the flavor, texture, and appearance are critical, the overall acceptance integrates a holistic evaluation of all these characteristics, reflecting the global perception of the product. The incorporation of nanoliposomes into yogurt significantly improves the overall acceptance of the yogurt compared to the control yogurt, which is an artisanal and natural yogurt without additives [86]. Based on the overall acceptance of the product, additional analyses are recommended to address various challenges in the development and distribution of yogurt in the food industry. It would be necessary to obtain regulatory approvals to ensure that nanoliposomes are safe for human consumption. Additionally, consumer perception needs to be addressed to overcome potential consumer reluctance towards products with nanotechnology by emphasizing safety and naturalness [86].

## 3. Materials and Methods

### 3.1. Chemical Reagents

All chemical reagents utilized in this study, including food-grade fucoxanthin (FXN), soy phosphatidylcholine (SPC), cholesterol, DPPH (1,1-diphenyl-2-picrylhydrazyl), ABTS [2,2′-azinobis (3-ethylbenzothiazoline)-6-sulfonic acid], dimethyl sulfoxide (DMSO), sodium acetate buffer, FeCl_3_ (ferric chloride), TPTZ (2,4,6-tripyridyl-s-triazine), Triton X-100, AAPH [2,2′-azobis (2-methylpropionamidine) dihydrochloride], and PBS (phosphate-buffered saline), were sourced from Sigma-Aldrich Co. (St. Louis, MO, USA). All other solvents used were of analytical grade and the highest commercial quality. Fucoxanthin used in this study was obtained from *Undaria pinnatifida*, a species of brown algae, and purchased as food-grade fucoxanthin from Sigma-Aldrich. The algae are cultivated, primarily in regions such as Asia, where seaweed farming practices contribute to sustainable resource management and align with the principles of the circular economy.

### 3.2. Biological Material and Ethical Considerations

All techniques involving human red blood cells (RBCs) were carried out in compliance with international regulations (FDA: CFR—Code of Federal Regulations Title 21, Part 640 for human blood and blood products, Subpart B Red Blood Cells, Sec. 640.14 Blood Tests [21 CFR 640.14]) and, in accordance with the Official Mexican Standard NOM-253-SSA1-2012 [87], which establishes guidelines for the collection, processing, and use of human blood and its components for therapeutic purposes. The RBCs’ membrane was donated by the clinical analysis laboratory of the University of Guadalajara, accredited by ISO-IEC 17025 (NMX-EC-17025) and ISO 15189 developed by the ISO/TC 212 technical committee (Clinical Laboratory Testing and In Vitro Diagnostic Systems), with ISO/IEC 17025 and ISO 9001 as reference standards [88,89,90,91]. Human red blood cells were collected from healthy adult volunteers (aged 20 to 40) containing approximately 4.7 to 6.1 × 10^6^ cells/μL. Before the procedure, informed consent was obtained from each individual. The venous puncture technique was applied to collect human red blood cells in a sterile vial with EDTA used as an anticoagulant. Using erythrocyte membranes as a cellular model aims to evaluate the erythroprotective potential of the yogurt formulation [92,93,94]. According to institutional procedure, study approval was obtained by the authors (CI 2023-47).

### 3.3. Synthesis of Fucoxanthin-Loaded Nanoliposomes

Fucoxanthin-loaded nanoliposomes (FXN-LN) were prepared using the ultrasonic film dispersion [26]. A quantity of 1 mg of fucoxanthin was dissolved in 5 mL of ethanol/sodium phosphate buffer (0.05 mol/L, pH 7.4). Lipid materials (SPC and Chol, 5:1 *w*/*w*) for the formation of liposomal vehicles were added to the fucoxanthin solution and dissolved into ethanol. This solution underwent rotary evaporation (Heidolph Laborata 4000, Schwabach, Germany) to completely remove the ethanol. Once the solvent was removed, the sample was hydrated with 25 mL of sodium phosphate buffer (0.05 mol/L, pH 7.4) and agitated for 30 min at 50 °C. Subsequently, the suspension underwent homogenization assisted by high-power ultrasonic pulses (Branson Digital Sonifier Qsonica, LLC. Branson, MI, USA), 15 s per pulse, three pulses with a one-minute rest interval at an amplitude of 55% at 400 w and 500 mHz to reduce the particular size. Finally, the nanoliposomes were freeze-dried to be later integrated into an amber glass bottle under a nitrogen atmosphere and stored at 4 °C before the study.

### 3.4. Morphological Study

The morphological study of FXN-LN was conducted using scanning electron microscopy (HT7700, Hitachi, Tokyo, Japan). The lyophilized fucoxanthin-loaded nanoliposomal carriers were placed onto a copper tape to form a film. The samples were then coated with gold and analyzed by SEM [26].

### 3.5. Encapsulation Efficiency

The encapsulation efficiency measurement was conducted using the extraction methodology outlined by Pan et al. [27]. A volume of 400 μL of FXN-LN solution was mixed with 1 mL of petroleum ether, followed by constant agitation (45 rpm) for 5 min at 30 °C. The resulting mixture was centrifuged at 4000× *g* for 5 min to recover the supernatant. The upper portion was collected and subjected to rotary evaporation to remove the petroleum ether. After evaporation, the residual sample was resuspended in chloroform. The content of free fucoxanthin was quantified at 460 nm using a 96-well microplate reader (UV-Vis spectrophotometer, Thermo Fisher Scientific Inc. Multiskan GO, New York, NY, USA). The encapsulation efficiency (%) was calculated using the following equation:

(2) $$Encapsulation\;Efficiency=\frac{{FXN}_{total}-{FXN}_{free}}{{FXN}_{total}}\times 100\;$$

### 3.6. Centrifugal Stability Measurement

The stability of nanoliposome samples was examined using the method of Ghorbanzade et al. [86] with slight modification. We subjected a volume of 5 mL of nanoliposomes to centrifugation at 3500× *g* for 15 min. Nanoliposome stability (NS) was calculated as follows:

(3) $$NS=\frac{F_{ev}}{I_{ev}}\times 100$$

where *F_ev_* is the final volume of the bottom phase and *I_ev_* is the initial volume of liposomal dispersion.

### 3.7. In Vitro Release

In vitro release studies were conducted using 10 mL of pure fucoxanthin solution and FXN-LN at the same concentration (1 mg). This volume was placed into a dialysis bag (8000–14,000 Da). The dialysis membrane was immersed in 100 mL of PBS (0.05 mol/L, pH 7.4) at 37 °C with constant agitation at 100 rpm. Aliquots of 1 mL were withdrawn from the release medium at intervals of 0.5, 1, 2, 4, 6, 8, 10, 12, and 24 h. The concentration of released fucoxanthin was measured in a microplate reader at a wavelength of 460 nm. The results will be reported as the percentage of fucoxanthin released. All release tests were conducted in triplicate [24]. The release rate of Ant was obtained using the following equation:

(4) $$Release\;rate=\frac{Released\;}{Total\;FXN}\times 100$$

### 3.8. Preparation of Potentially Functional Yogurt

**Figure 12:**
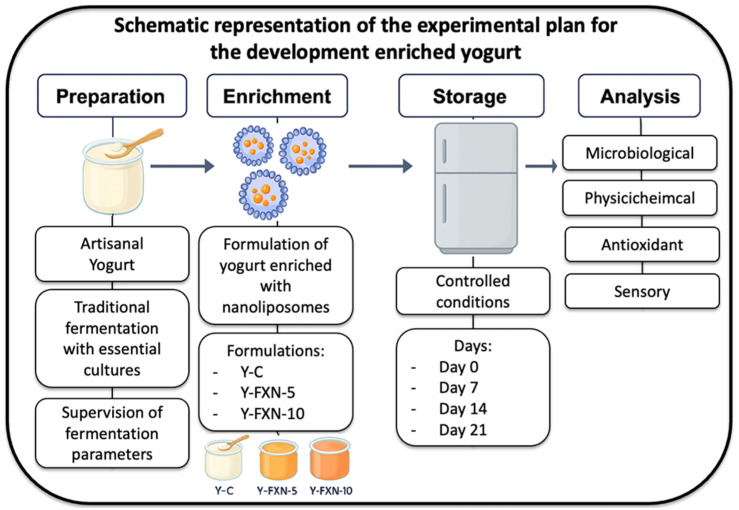
Schematic representation of the experimental design for the development and evaluation of fucoxanthin-loaded nanoliposome-enriched yogurt.

The experimental plan, presented in graphical format for the development and evaluation of yogurt enriched with fucoxanthin-loaded nanoliposomes is shown in Figure 12, which provides a visual summary of the experimental workflow. The study in this section was structured into five main stages: (i) preparation of the artisanal yogurt base; (ii) synthesis and incorporation of fucoxanthin-loaded nanoliposomes; (iii) enrichment of the yogurt; (iv) cold storage under controlled conditions; and (v) multiparametric analysis to evaluate the impact of enrichment on various product properties.

This schematic representation facilitates a clear and organized understanding of the experimental strategy by visually integrating the treatment structure, the sequential phases of the process, and the analytical workflow implemented throughout this study.

#### 3.8.1. Preparation and Formulation

Artisanal yogurt was obtained from a local store in Ameca, Jalisco, Mexico. It is produced through the fermentation of milk using live bacterial cultures, resulting in a fresh product with authentic flavor and creamy texture. The yogurt used in this study was prepared with traditional starter cultures consisting of *Lactobacillus delbrueckii* subsp. *bulgaricus* and *Streptococcus thermophilus*, the essential microorganisms required for yogurt production according to international standards. These microorganisms are responsible for developing the characteristic acidity, texture, and sensory profile of yogurt and may also contribute potential probiotic benefits. All experiments were performed in triplicate using three independent batches of artisanal yogurt, prepared by the same local supplier under previously established standardized conditions. The research team directly supervised the yogurt production to ensure consistent fermentation parameters (time, temperature, type of starter cultures, and their proportions). This strategy was implemented to guarantee the reproducibility of the process prior to the incorporation of fucoxanthin-loaded nanoliposomes.

This yogurt is valued for its high nutritional content, being free from preservatives and artificial additives, and its digestive health benefits due to probiotics. Two formulations of yogurt enriched with 5 and 10% nanoliposomes loaded with fucoxanthin were prepared in separate 100 g yogurt samples [86]. The formulations were stored in glass containers in a refrigerator at 4 °C for subsequent analysis. The yogurt enriched with nanoliposomes was placed in airtight containers to prevent exposure to air and light. The containers were stored at 4 °C, simulating refrigeration conditions for 21 days. Samples of the enriched yogurt were taken as part of periodic sampling during the storage period every 7 days. Under this condition, the effect of cold storage conditions on the stability and physicochemical and rheological characteristics of the formulations can systematically be evaluated, providing important information for their application in refrigerated food products.

#### 3.8.2. Chemical Characterization

Proximate analyses were performed on samples of artisanal yogurt (control) and yogurt enriched with fucoxanthin-loaded nanoliposomes to determine their dry matter, humanity, protein, lipid, ash, and carbohydrate content. These determinations were performed following the methodology established by AOAC Method 925.23 (Association of Official Analytical Chemists) [95]. All analyses were conducted in triplicate for both the control group and the enriched yogurt.

#### 3.8.3. Microbiological Quality of Yogurt

Microbiological analyses were performed on the yogurt samples prior to sensory evaluation to ensure their safety and sanitary quality. The levels of total mesophilic aerobes, total coliforms, fecal coliforms, molds, and yeasts were determined in accordance with the guidelines established by NOM-243-SSA1-2010 for fermented dairy products. The samples were inoculated onto selective media and incubated under standard conditions. All formulations analyzed complied with the microbiological limits permitted for dairy products, ensuring that the samples were suitable for human consumption during the sensory analysis [7].

### 3.9. Effect of Cold Storage Conditions on the Antioxidant, Physicochemical, and Rheological Properties of Yogurt Enriched with FXN-LN

#### 3.9.1. Antioxidant Properties

The measurement of the antioxidant properties of fucoxanthin-loaded nanoliposome was carried out for 21 days under cold storage. The antioxidant activity was evaluated using three methods. The FRAP assay, based on Benzie and Strain [96], involved preparing a working solution from sodium acetate buffer, triazine-TPTZ in HCl, and ferric chloride, and measuring the reduction of TPTZ at 638 nm. The DPPH• assay followed 96. Brand-Williams et al. [97] required dissolving DPPH• in ethanol, adjusting its absorbance, and mixing it with the sample to measure absorbance at 515 nm. The ABTS assay, modified from Re et al. [98], involved preparing an ABTS+• solution with potassium persulfate, adjusting its absorbance, and mixing it with the sample to measure absorbance at 734 nm. All assays were performed in triplicate, and measurements were taken using a microplate reader (Thermo Fisher Scientific Inc. Multiskan GO, New York, NY, USA).

#### 3.9.2. Erythroprotective Potential

The evaluation of erythroprotective potential was conducted through a series of assays assessing oxidative inhibition by AAPH-induced hemolysis, ultraviolet radiation-induced hemolysis, and membrane stabilization test (heat-induced hemolysis inhibition and hypotonicity-induced hemolysis inhibition). The antihemolytic activity was evaluated by inducing oxidative hemolysis using the free-radical generator AAPH [2,2′-azobis-(2-methylpropionamidine)], following the methods of Ruiz-Cruz et al. [93]. Human erythrocytes were obtained from healthy adults aged 20–45 years who were informed about the procedure and provided informed consent. A 2% erythrocyte suspension was prepared, and an AAPH solution was made at 40 mM (pH 7.4). The reaction was initiated by mixing 100 μL of erythrocytes (2%), 100 μL of the sample, and 100 μL of AAPH. Controls were prepared as follows: 100 μL of erythrocytes + 100 μL of PBS + 100 μL of AAPH (positive control, hemolysis) and 100 μL of erythrocytes + 200 μL of PBS (negative control, no hemolysis). Samples and controls were incubated at 37 °C for 3 h with constant shaking at 45 rpm. After incubation, 1 mL of PBS was added to each sample, including the controls, and they were centrifuged at 1500 rpm for 10 min. Finally, 300 µL of supernatant was collected, and the hemoglobin released by oxidation was quantified at 540 nm using a 96-well microplate reader (Multiskan Go, Thermo Scientific, Waltham, MA, USA).

Ultraviolet radiation-induced photo-oxidation was carried out using UV-A (315–395 nm) and UV-B (280–315 nm) light at an intensity of 0.85 mW/cm^2^, with the UV light chamber maintained at 18 ± 1 °C. The photoprotective effect of fucoxanthin against UV-induced peroxidation was evaluated. Fucoxanthin was dissolved in DMSO and diluted in PBS:DMSO (90:10). A 1% erythrocyte suspension was used, with 3 mL aliquots placed in sterile vials and 150 µL of the sample added. Positive controls were erythrocytes exposed to UV radiation, and negative controls were non-irradiated erythrocytes. Samples were pre-incubated at 37 °C for 30 min, then exposed to UV-A and UV-B for 0, 15, 30, 60, and 120 min at 10 cm from the lamps. Post-irradiation, samples were centrifuged at 2000× *g* for 10 min, and 300 µL of each sample was placed in a 96-well microplate for measurement at 540 nm [4].

Heat-induced hemolysis and hypotonicity-induced hemolysis assays were conducted to evaluate various tested yogurt formulations’ membrane stabilization (%). Encapsulated fucoxanthin carried in yogurt stabilizes erythrocyte membranes by inhibiting the release of hemoglobin constituents, making it a useful control in the Membrane-Stabilizing Capacity Assay to assess the potential of antioxidants as erythroprotective agents. The heat-induced hemolysis assay followed Agarwal et al.’s [99] methodology with minor modifications, involving the incubation of erythrocyte suspensions and samples at 55 °C for 30 min, followed by PBS addition and centrifugation, with absorbance measured at 540 nm. The hypotonicity-induced hemolysis assay followed Agarwal et al.’s [99] modified methodology, using distilled water as the hypotonic solution, and involved a similar incubation and centrifugation process, with different controls used for comparison.

#### 3.9.3. Physicochemical Properties

##### Electrical Conductivity

Measurements of the electrical conductivity of the yogurt samples were carried out using the method described in Alimentarius, Codex [100] with slight modifications: the samples were diluted by 1:2 with distilled water, measured at 25 °C, and a platinum electrode was used. The conductivity meter was calibrated with a standard solution before each series of measurements, and the readings were taken after a 2 min stabilization period.

##### pH

The pH was assessed using a digital pH meter (HI 2211 PH/MV, HANNA) calibrated before measurement with reference buffer solutions (pH 4.0 and 7.0), according to AOCS [101,102], by immersing an electrode in the solution. The sample (1 g) was mixed in 10 mL of distilled water for analysis, and the reading was recorded.

##### Titratable Acidity

Titratable acidity and pH were measured according to the official 942.15 method [101,102]. Titratable acidity was expressed as grams of lactic acid per 100 g of product after mixing 10 g of yogurt sample with 10 mL of hot distilled water and titrating with 0.1 N NaOH using a 0.5% phenolphthalein indicator.

##### Syneresis Susceptibility (STS)

To determine the syneresis of different yogurt formulations, 20 g of yogurt was centrifuged at 2500× *g* for 5 min. The separated liquid was collected in a graduated cylinder [103]. The percentage of syneresis was calculated using the following equation:

(5) $$Syneresis\;\left(\%\right)=\frac{Total\;weight\;of\;liquid\;separated\;}{Total\;yogurt\;weight}\times 100\;$$

##### Water-Holding Capacity (WHC)

To determine water-holding capacity (WHC), 2.5 g of sample were taken [91] and homogenized with 30 mL of distilled water at 30 °C in a water bath for 30 min. The mixture was then centrifuged at 3000× *g* for 30 min. The supernatant was decanted and dried in an oven (3489 M-1, Barnstead International, Dubuque, IA, USA) at 90 °C for 24 h. Additionally, the weight of the precipitate in the cylinders was recorded. The WHC results were expressed as a percentage of water-holding capacity [104].

##### Texture

The texture of the yogurt was evaluated using a TA-XT2 texture analyzer with Exponent software, version 6.1 (Stable Micro Systems, Godalming, UK) with accompanying software. The probe utilized was cylindrical with a diameter of 25 mm (P25/L), and the test conditions included a pre-test speed of 5 mm/s, a test speed of 3.0 mm/s, a target mode set to strain, a duration of 3 s, and a trigger force of 0.5 g using a 2000 g calibration weight. The test was conducted directly on a 40 g sample cup and performed in triplicate. All experiments were carried out at a temperature of 5 °C. The primary textural properties of the yogurt were measured, including firmness or gel strength (peak compression force during penetration) and adhesiveness (negative force area) [54].

#### 3.9.4. Rheological Analysis

Measurements were conducted using a rotational viscometer (Thermo Haake DC 10, model VT 550, Karlsruhe, Germany), with concentric cylinders (NV ST 807-0713 CE and NV 807-0702), and the data were collected by the Pro Rheowin software program © (version 2.93, Haake). These analyses were performed in triplicate. The temperature was 25 °C, and the shear rate ranged from 0 to 2000 s^−1^ (upward curve) and from 2000 to 0 s^−1^ (downward curve); each curve was obtained over 3 min. The flow behavior was described using the Power Law model, and the thixotropic behavior of the yogurt was evaluated by calculating the area of the hysteresis loop between the upward and downward flow curves [71].

#### 3.9.5. Sensory Analysis

Sensory evaluation was carried out according to the guidelines and as mentioned in Lawless et al. [104] and Stone and Sidel [105]. Thirty panelists aged between 20 and 40 years were selected to participate in the sensory panel. The sensory evaluation was conducted at the Department of Medical and Life Sciences facilities, Cienega University Center (CUCIÉNEGA), University of Guadalajara, Av. Universidad 1115, Lindavista, 47,820 Ocotlán, Jalisco, Mexico. The sensory evaluation (color, flavor, aftertaste, scent, consistency, texture, appearance, and overall acceptance) were based on 10-point hedonic scales (0: extremely dislike to 9: extremely like). Each sample was individually rated, and the samples were presented to the panelists in individual plastic containers. The yogurts (coded with 3 digits) were randomly presented to the panel group in each session [103,104].

### 3.10. Statistical Analysis

The results were reported as means ± standard deviation (SD) of at least three repetitions (*n* > 3). All data were analyzed using JMP software v16 for Mac and expressed as mean ± SD (standard deviation). One-way and two-way ANOVA were performed to explore the interaction between the evaluated factors. Tukey’s test was applied with a confidence level of *p* < 0.05.

## 4. Conclusions

The nanoliposomal vehicles that encapsulated the fucoxanthin developed in this study were produced by applying a simple, low-cost ultrasonic film dispersion technique that could be scaled up industrially. Incorporating fucoxanthin-loaded nanoliposomes into yogurt has revealed significant findings about the bioactive potential to enhance and increase the therapeutic properties of yogurt. The nanoliposomes showed high encapsulation efficiency and stability, allowing for the controlled and sustained release of fucoxanthin. This encapsulation method protects fucoxanthin from degradation during storage and increases its solubility and bioavailability. Fucoxanthin nanoliposome-enriched yogurt formulations demonstrated significant improvements in the antioxidant capacity. It was confirmed that yogurt with nanoliposomes retained higher antioxidant activity without being affected by cold storage compared to yogurt without enrichment. On the other hand, although the incorporation of nanoliposomes decreases the viscosity and firmness of yogurt, which could affect the texture of the product, an improvement in the pH stability and titratable acidity was observed. Furthermore, syneresis was reduced while the water retention capacity increased, improving the product stability during storage. The formulation with 10% fucoxanthin nanoliposomes not only improved the antioxidant properties of yogurt, but also positively impacted sensory properties such as the color, taste, and overall acceptance, suggesting a higher likelihood of consumer acceptance. Incorporating fucoxanthin-loaded nanoliposomes into yogurt could be a viable strategy to enhance the potential functionality and stability of the product. However, challenges related to texture need to be addressed to optimize the formulation fully. These findings create opportunities for future research on optimizing the interaction between nanoliposomes and the yogurt matrix and exploring other bioactive compounds encapsulated in nanoliposomes for applications in functional foods. Finally, these results suggest that nanoliposomal vehicles are suitable for carrying fucoxanthin. Their incorporation into food matrices is critical to developing functional foods. Regulatory approvals and consumer perceptions regarding nanotechnology-based products must be addressed, emphasizing their safety and health benefits.

## Figures and Tables

**Table 1:** Proximate analysis of yogurt enriched with fucoxanthin-loaded nanoliposomes.

			Total Dry Weight			
Samples	Dry Matter (%)	Humidity (%)	Protein (%)	Fat (%)	CHO (%)	Ashes (%)
Y-C	13.5 ^a^ ± 1.56	86.5 ^a^ ± 2.2	37.3 ^a^ ± 1.3	31.6 ^a^ ± 2.0	24.9 ^a^ ± 1.9	6.5 ^a^ ± 0.3
Y-FXN-5	14.2 ^a^ ± 1.32	85.8 ^a^ ± 2.4	36.4 ^a^ ± 2.0	33.5 ^a^ ± 2.6	22.8 ^a^ ± 1.2	7.2 ^a^ ± 0.2
Y-FXN-10	14.7 ^a^ ± 2.75	85.3 ^a^ ± 2.6	35.9 ^a^ ± 1.6	34.1 ^a^ ± 1.7	22.2 ^a^ ± 1.2	7.9 ^a^ ± 0.5

The values represent mean averages. No statistically significant differences (same superscript a) were observed among the samples according to the post hoc test (one-way ANOVA, *p* > 0.05). Data are presented as the mean of three replicates. Mean ± standard deviation (SD) of *n* = 3. Y-C = Control Yogurt without nanocapsules; Y-FXN-5 = Yogurt enriched with 5% nanocapsules; Y-FXN-10 = Yogurt enriched with 10% nanocapsules.

**Table 2:** Effect of cold storage on the antioxidant properties (DPPH, ABTS, and FRAP) of yogurt formulations added with nanoliposomes loaded with fucoxanthin.

Samples	Storage Time (Days)	Free-Radical Scavenging (%)		mmol TE/g
		DPPH•	ABTS+•	FRAP
Y-C	0	24.8 ^e^ ± 1.3	33.72 ^f^ ± 1.8	2.9 ^c^ ± 0.1
	7	24.3 ^e^ ± 2.5	30.4 ^fg^ ± 2.4	2.4 ^d^ ± 0.1
	14	21.7 ^f^ ± 1.7	28.41 ^g^ ± 2.9	2.2 ^d^ ± 0.1
	21	19.2 ^f^ ± 2.8	25.9 ^g^ ± 1.2	2.3 ^d^ ± 0.1
Y-FXN-5	0	35.0 ^c^ ± 0.9	43.4 ^d^ ± 1.6	3.1 ^ab^ ± 0.1
	7	34.1 ^c^ ± 1.8	41.6 ^de^ ± 1.7	3.2 ^a^ ± 0.1
	14	32.6 ^cd^ ± 2.8	39.3 ^e^ ± 1.6	3.0 ^b^ ± 0.1
	21	30.2 ^d^ ± 1.6	39.6 ^e^ ± 2.9	2.9 ^b^ ± 0.1
Y-FXN-10	0	52.9 ^a^ ± 1.2	97.9 ^a^ ± 2.9	3.2 ^a^ ± 0.1
	7	50.2 ^b^ ± 0.4	92.5 ^b^ ± 2.3	3.1 ^a^ ± 0.1
	14	51.3 ^ab^ ± 1.6	90.2 ^bc^ ± 1.2	3.2 ^a^ ± 0.1
	21	50.3 ^b^ ± 1.2	88.4 ^c^ ± 0.7	3.0 ^b^ ± 0.1

The results are expressed as mean ± standard deviation (*n* = 3). Means with different lowercase letters (a–g: time effects) within the same treatment are significantly different. Y-C = Control Yogurt without nanocapsules; Y-FXN-5 = Yogurt enriched with 5% nanocapsules; Y-FXN-10 = Yogurt enriched with 10% nanocapsules.

**Table 3:** Erythroprotective potential of yogurt formulations that were added with nanoliposomes loaded with fucoxanthin.

Samples	HI (%)	PHI (%)	Heat-IH	Hypo-IH
Y-C	17.3 ^c^ ± 2.7	61.07 ^b^ ± 2.5	25.50 ^b^ ± 2.4	81.46 ^b^ ± 1.2
Y-FXN-5	63.8 ^b^ ± 2.3	54.93 ^c^ ± 1.9	25.17 ^b^ ± 2.9	80.87 ^b^ ± 2.9
Y-FXN-10	82.4 ^a^ ± 1.5	82.40 ^a^ ± 2.6	46.80 ^a^ ± 3.6	93.62 ^a^ ± 2.2

The results are expressed as mean ± standard deviation (*n* = 3). Means with different lowercase letters (a–c: time effects) within the same treatment are significantly different. Y-C = Control Yogurt without nanocapsules; Y-FXN-5 = Yogurt enriched with 5% nanocapsules; Y-FXN-10 = Yogurt enriched with 10% nanocapsules. HI: Hemolysis inhibition; PHI: Photohemolysis inhibition. Heat-IH: Inhibition of heat-induced hemolysis; Hypo-IH: Inhibition of hypotonicity-induced hemolysis.

**Table 4:** Effect of nanoencapsulation on the sensory characteristics of fucoxanthin-enriched yogurt.

Sensory Quality Attributes	Treatments		
	Y-C	Y-FXN-5	Y-FXN-10
Color	8.56 ^a^ ± 0.16	7.491 ^a^ ± 0.26	8.73 ^a^ ± 0.47
Flavor	7.83 ^a^ ± 0.23	7.64.73 ^b^ ± 0.42	8.09 ^c^ ± 0.83
Aftertaste	7.24 ^b^ ± 0.26	8.00 ^a^ ± 0.25	8.18 ^a^ ± 1.08
Scent	8.61 ^a^ ± 0.45	7.73 ^ab^ ± 0.45	8.64 ^b^ ± 0.67
Consistency	8.29 ^a^ ± 0.35	7.55 ^ab^ ± 0.34	8.19 ^b^ ± 0.94
Texture	8.22 ^a^ ± 0.23	7.91 ^a^ ± 0.65	8.36 ^a^ ± 0.92
Appearance	8.29 ^a^ ± 0.32	7.64 ^a^ ± 0.32	8.82 ^b^ ± 0.40
General acceptance	7.88 ^a^ ± 0.15	8.00 ^a^ ± 0.21	8.36 ^a^ ± 0.81

Means with different lowercase letters (a–c: yogurt formulations) within the same sensory quality attributes are significantly different. Sensory scale: 0 being very unpleasant and 9 being very pleasant. Values in the same column with different letters are significantly different. Y-C: control sample (unfortified yogurt); Y-FXN-5: 5% fucoxanthin-enriched yogurt; Y-FXNt-10: 10% fucoxanthin-enriched yogurt.

## Data Availability

The data supporting the findings of this research are provided within this article. Additional details can be obtained from the corresponding authors upon request.

## References

[1] Rashwan A.K., Osman A.I., Chen W. (2023). Natural Nutraceuticals for Enhancing Yogurt Properties: A Review. Environ. Chem. Lett..

[2] Alkobeisi F., Varidi M.J., Varidi M., Nooshkam M. (2022). Quinoa Flour as a Skim Milk Powder Replacer in Concentrated Yogurts: Effect on Their Physicochemical, Technological, and Sensory Properties. Food Sci. Nutr..

[3] Almutairi B., Turner M.S., Fletcher M.T., Sultanbawa Y. (2021). The Impact of Commercial Prebiotics on the Growth, Survival and Nisin Production by *Lactococcus lactis* 537 in Milk. LWT.

[4] González-Vega R.I., Robles-García M.Á., Mendoza-Urizabel L.Y., Cárdenas-Enríquez K.N., Ruiz-Cruz S., Gutiérrez-Lomelí M., Iturralde-García R.D., Avila-Novoa M.G., Villalpando-Vargas F.V., Del-Toro-Sánchez C.L. (2023). Impact of the ABO and RhD Blood Groups on the Evaluation of the Erythroprotective Potential of Fucoxanthin, β-Carotene, Gallic Acid, Quercetin and Ascorbic Acid as Therapeutic Agents against Oxidative Stress. Antioxidants.

[5] Peng J., Yuan J.-P., Wu C.-F., Wang J.-H. (2011). Fucoxanthin, a Marine Carotenoid Present in Brown Seaweeds and Diatoms: Metabolism and Bioactivities Relevant to Human Health. Mar. Drugs.

[6] Lourenço-Lopes C., Fraga-Corral M., Jimenez-Lopez C., Carpena M., Pereira A.G., Garcia-Oliveira P., Prieto M.A., Simal-Gandara J. (2021). Biological Action Mechanisms of Fucoxanthin Extracted from Algae for Application in Food and Cosmetic Industries. Trends Food Sci. Technol..

[7] FAO (2022). The State of World Fisheries and Aquaculture 2022: Towards Blue Transformation.

[8] Kakehi S., Onitsuka G., Kidokoro H., Mimura N., Takewaka S. (2025). Impact on Brown Macroalga *Undaria pinnatifida* Farming under Changing Ocean Climate. Climate Change Impacts and Adaptation Strategies in Japan.

[9] Rostamabadi H., Reza Falsafi S., Mahdi Jafari S. (2019). Nanoencapsulation of Carotenoids within Lipid-Based Nanocarriers. J. Control. Release.

[10] Sun Y., Chi J., Ye X., Wang S., Liang J., Yue P., Xiao H., Gao X. (2021). Nanoliposomes as Delivery System for Anthocyanins: Physicochemical Characterization, Cellular Uptake, and Antioxidant Properties. LWT.

[11] Bhosale S., Fulpagare Y.G., Desale R.J. (2019). Nanoliposomes: Applications in Food and Dairy Industry. Int. J. Adv. Res. Biol. Sci..

[12] Cheng X., Zang M., Wang S., Zhao X., Zhai G., Wang L., Li X., Zhao Y., Yue Y. (2022). Physicochemical and Antioxidant Properties of Nanoliposomes Loaded with Rosemary Oleoresin and Their Oxidative Stability Application in Dried Oysters. Bioengineering.

[13] Barkallah M., Dammak M., Louati I., Hentati F., Hadrich B., Mechichi T., Ayadi M.A., Fendri I., Attia H., Abdelkafi S. (2017). Effect of *Spirulina platensis* Fortification on Physicochemical, Textural, Antioxidant and Sensory Properties of Yogurt during Fermentation and Storage. LWT.

[14] da Silva D.F., Junior N.N.T., Gomes R.G., dos Santos Pozza M.S., Britten M., Matumoto-Pintro P.T. (2017). Physical, Microbiological and Rheological Properties of Probiotic Yogurt Supplemented with Grape Extract. J. Food Sci. Technol..

[15] Anuyahong T., Chusak C., Adisakwattana S. (2020). Incorporation of anthocyanin-rich riceberry rice in yogurts: Effect on physicochemical properties, antioxidant activity and in vitro gastrointestinal digestion. LWT.

[16] Megrous S., Al-Dalali S., Yang Z. (2024). Physicochemical and functional properties of yoghurt supplemented with bioactive low-molecular-weight casein hydrolysates. Int. Dairy J..

[17] Lee W.J., Lucey J.A. (2010). Formation and physical properties of yogurt. Asian-Australas. J. Anim. Sci..

[18] Oh N.S., Lee J.Y., Joung J.Y., Kim K.S., Shin Y.K., Lee K.-W., Kim S.H., Oh S., Kim Y. (2016). Microbiological characterization and functionality of set-type yogurt fermented with potential prebiotic substrates *Cudrania tricuspidata* and *Morus alba* L. leaf extracts. J. Dairy Sci..

[19] Mani-López E., Palou E., López-Malo A. (2014). Probiotic viability and storage stability of yogurts and fermented milks prepared with several mixtures of lactic acid bacteria. J. Dairy Sci..

[20] Liu D., Lv X.X. (2019). Effect of blueberry flower pulp on sensory, physicochemical properties, lactic acid bacteria, and antioxidant activity of set-type yogurt during refrigeration. J. Food Process. Preserv..

[21] Liu X.T., Zhang H., Wang F., Luo J., Guo H.Y., Ren F.Z. (2014). Rheological and structural properties of differently acidified and renneted milk gels. J. Dairy Sci..

[22] Brodziak A., Krol J., BarÇowska J., Teter A., Florek M. (2020). Changes in the physicochemical parameters of yoghurts with added whey protein in relation to the starter bacteria strains and storage time. Animals.

[23] Hu F., Liu W., Yan L., Kong F., Wei K. (2019). Optimization and characterization of poly(lactic-co-glycolic acid) nanoparticles loaded with astaxanthin and evaluation of anti-photodamage effect in vitro. R. Soc. Open Sci..

[24] Wang Q., Zhao Y., Guan L., Zhang Y., Dang Q., Dong P., Li J., Liang X. (2017). Preparation of astaxanthin-loaded DNA/chitosan nanoparticles for improved cellular uptake and antioxidation capability. Food Chem..

[25] Liu C., Zhang S., McClements D.J., Wang D., Xu Y. (2019). Design of astaxanthin-loaded core-shell nanoparticles consisting of chitosan oligosaccharides and poly(lactic-co-glycolic acid): Enhancement of water solubility, stability, and bioavailability. J. Agric. Food Chem..

[26] Tamjidi F., Shahedi M., Varshosaz J., Nasirpour A. (2014). Design and characterization of astaxanthin-loaded nanostructured lipid carriers. Innov. Food Sci. Emerg. Technol..

[27] Pan L.H., Liu F., Luo S.Z., Luo J.P. (2019). Pomegranate juice powder as sugar replacer enhanced quality and function of set yogurts: Structure, rheological property, antioxidant activity and in vitro bioaccessibility. LWT.

[28] Rodriguez-Ruiz V., Salatti-Dorado J.Á., Barzegari A., Nicolas-Boluda A., Houaoui A., Caballo C., Caballero-Casero N., Sicilia D., Venegas J.B., Pauthe E. (2018). Astaxanthin-loaded nanostructured lipid carriers for preservation of antioxidant activity. Molecules.

[29] Taksima T., Limpawattana M., Klaypradit W. (2015). Astaxanthin encapsulated in beads using ultrasonic atomizer and application in yogurt as evaluated by consumer sensory profile. LWT.

[30] Mozafari M.R., Weissig V. (2010). Nanoliposomes: Preparation and Analysis. Liposomes: Methods in Molecular Biology.

[31] Mozafari M.R. (2006). Nanocarrier Technologies: Frontiers of Nanotherapy.

[32] Wang Y., Lu Z., Wu H., Lv F. (2015). The Stability, Sustained Release and Cellular Antioxidant Activity of Curcumin Nanoliposomes. Molecules.

[33] Domoto N., Koenen M.E., Havenaar R., Mikajiri A., Chu B.S. (2013). The Bioaccessibility of Eicosapentaenoic Acid Was Higher from Phospholipid Food Products than from Mono and Triacylglycerol Food Products in a Dynamic Gastrointestinal Model. Food Sci. Nutr..

[34] Sahin O.I., Dundar A.N., Ozdemir S., Uzuner K., Parlak M.E., Dagdelen A.F., Saricaoglu F.T. (2022). Nanophytosomes as a Protection System to Improve the Gastrointestinal Stability and Bioavailability of Phycocyanin. Food Biosci..

[35] Mahfoudhi N., Ksouri R., Hamdi S. (2016). Nanoemulsions as Potential Delivery Systems for Bioactive Compounds in Food Systems: Preparation, Characterization, and Applications in Food Industry. Nanotechnology in the Agri-Food Industry.

[36] McClements D.J. (2018). Encapsulation, Protection, and Delivery of Bioactive Proteins and Peptides Using Nanoparticle and Microparticle Systems: A Review. Adv. Colloid Interface Sci..

[37] Li S., Lo C.-Y., Pan M.-H., Lai C.-S., Ho C.-T. (2013). Black Tea: Chemical Analysis and Stability. Food Funct..

[38] Le T.T., Phan T.T.Q., Van Camp J., Dewettinck K., Park Y.W. (2015). Milk and dairy polar lipids: Occurrence, purification, and nutritional and technological properties. Milk and Dairy Products in Human Nutrition.

[39] Aguilar-Pérez K.M., Medina D.I., Narayanan J., Parra-Saldívar R., Iqbal H.M.N. (2021). Synthesis and Nano-Sized Characterization of Bioactive Oregano Essential Oil Molecule-Loaded Small Unilamellar Nanoliposomes with Antifungal Potentialities. Molecules.

[40] Matos J., Afonso C., Cardoso C., Serralheiro M.L., Bandarra N.M. (2021). Yogurt Enriched with *Isochrysis galbana*: An Innovative Functional Food. Foods.

[41] Peng C.H., Chang C.H., Peng R.Y., Chyau C.C. (2010). Improved membrane transport of astaxanthin by liposomal encapsulation. Eur. J. Pharm. Biopharm..

[42] Risaliti L., Kehagia A., Daoultzi E., Lazari D., Bergonzi M.C., Vergkizi-Nikolakaki S., Bilia A.R. (2019). Liposomes loaded with Salvia triloba and Rosmarinus officinalis essential oils: In vitro assessment of antioxidant, antiinflammatory and antibacterial activities. J. Drug Deliv. Sci. Technol..

[43] Hammoud Z., Gharib R., Fourmentin S., Elaissari A., Greige-Gerges H. (2019). New findings on the incorporation of essential oil components into liposomes composed of lipoid S100 and cholesterol. Int. J. Pharm..

[44] Hamad I., Harb A.A., Bustanji Y. (2024). Liposome-Based Drug Delivery Systems in Cancer Research: An Analysis of Global Landscape Efforts and Achievements. Pharmaceutics.

[45] Acurio Arcos L.P., Cadena Masabanda W.A. (2024). Physicochemical, Rheological, Sensory, and Proximal Properties of Yogurt Flavored with Aloe vera Gel. Emir. J. Food Agric..

[46] Ministry of Health (Mexico) (2010). Official Mexican Standard NOM-243-SSA1-2010: Products and Services. Milk, Milk Formula, Combined Dairy Products and Dairy Derivatives. Sanitary Provisions and Specifications. Test Methods. https://www.fao.org/faolex/results/details/en/c/LEX-FAOC097698/.

[47] Sarıtaş S., Portocarrero A.C.M., Miranda López J.M., Lombardo M., Koch W., Raposo A., El-Seedi H.R., de Brito Alves J.L., Esatbeyoglu T., Karav S. (2024). The Impact of Fermentation on the Antioxidant Activity of Food Products. Molecules.

[48] Corrêa R.C.G., Barros L., Fernandes Â., Sokovic M., Bracht A., Peralta R.M., Ferreira I.C.F.R. (2018). A natural food ingredient based on ergosterol: Optimization of the extraction from Agaricus blazei, evaluation of bioactive properties and incorporation in yogurts. Food Funct..

[49] Bourne M.C. (2002). Food Texture and Viscosity: Concept and Measurement.

[50] Lee J.-W., Lucey J.A. (2006). Impact of Gelation Conditions and Structural Breakdown on the Physical and Sensory Properties of Stirred Yogurts. J. Dairy Sci..

[51] Jeong C.H., Ryu H., Zhang T., Lee C.H., Seo H.G., Han S.G. (2018). Green Tea Powder Supplementation Enhances Fermentation and Antioxidant Activity of Set-Type Yogurt. Food Sci. Biotechnol..

[52] Zahoor I., Allai F.M., Ahmad S., Al-Shabib N. (2020). Food Antioxidants: Functional Aspects and Preservation During Food Processing. Functional Food Products and Sustainable Health.

[53] Gómez-Estaca J., Balaguer M.P., López-Carballo G., Gavara R., Hernández-Muñoz P. (2017). Improving Antioxidant and Antimicrobial Properties of Curcumin by Means of Encapsulation in Gelatin through Electrohydrodynamic Atomization. Food Hydrocoll..

[54] Kristl J., Teskac K., Caddeo C., Abramovic Z., Sentjurc M. (2009). Improvements of Cellular Stress Response on Resveratrol in Liposomes. Eur. J. Pharm. Biopharm..

[55] Abd El-Emam M.M., Mostafa M., Farag A.A., Youssef H.S., El-Demerdash A.S., Bayoumi H., Gebba M.A., El-Halawani S.M., Saleh A.M., Badr A.M. (2023). The Potential Effects of Quercetin-Loaded Nanoliposomes on Amoxicillin/Clavulanate-Induced Hepatic Damage: Targeting the SIRT1/Nrf2/NF-Œ∫B Signaling Pathway and Microbiota Modulation. Antioxidants.

[56] Wu Y., Wang K., Liu Q., Liu X., Mou B., Lai O.-M., Tan C.-P., Cheong L.-Z. (2022). Selective Antibacterial Activities and Storage Stability of Curcumin-Loaded Nanoliposomes Prepared from Bovine Milk Phospholipid and Cholesterol. Food Chem..

[57] Hasan M., Elkhoury K., Kahn C.J.F., Arab-Tehrany E., Linder M. (2019). Preparation, Characterization, and Release Kinetics of Chitosan-Coated Nanoliposomes Encapsulating Curcumin in Simulated Environments. Molecules.

[58] Linder M., Arab-Tehrany E. (2018). The Positive Role of Curcumin-Loaded Salmon Nanoliposomes on the Culture of Primary Cortical Neurons. Mar. Drugs.

[59] Yakoubi S., Kobayashi I., Uemura K., Nakajima M., Isoda H., Ksouri R., Saidani-Tounsi M., Neves M.A. (2021). Essential-Oil-Loaded Nanoemulsion Lipidic-Phase Optimization and Modeling by Response Surface Methodology (RSM): Enhancement of Their Antimicrobial Potential and Bioavailability in Nanoscale Food Delivery System. Foods.

[60] Chen Y., He N., Yang T., Cai S., Zhang Y., Lin J., Huang M., Chen W., Zhang Y., Hong Z. (2022). Fucoxanthin Loaded in Palm Stearin- and Cholesterol-Based Solid Lipid Nanoparticle-Microcapsules, with Improved Stability and Bioavailability In Vivo. Mar. Drugs.

[61] Sridhar K., Inbaraj B.S., Chen B.-H. (2021). Recent Advances on Nanoparticle Based Strategies for Improving Carotenoid Stability and Biological Activity. Antioxidants.

[62] Verardi A., Sangiorgio P., Lopresto C.G., Casella P., Errico S. (2023). Enhancing Carotenoids’ Efficacy by Using Chitosan-Based Delivery Systems. Nutraceuticals.

[63] Nuñez de González M.T., Attaie R., Woldesenbet S., Mora-Gutierrez A., Jung Y. (2023). Fucoxanthin as a Biofunctional Compound in Goat Milk Yogurt: Stability and Physicochemical Effects. Fermentation.

[64] Mok I.K., Lee J.K., Kim J.H., Pan C.H., Kim S.M. (2018). Fucoxanthin Bioavailability from Fucoxanthin-Fortified Milk: In Vivo and In Vitro Study. Food Chem..

[65] Mok I.K., Yoon J.R., Pan C.H., Kim S.M. (2016). Development, Quantification, Method Validation, and Stability Study of a Novel Fucoxanthin-Fortified Milk. J. Agric. Food Chem..

[66] Gürbüz Z., Erkaya-Kotan T., Şengül M. (2021). Evaluation of Physicochemical, Microbiological, Texture and Microstructure Characteristics of Set-Style Yoghurt Supplemented with Quince Seed Mucilage Powder as a Novel Natural Stabiliser. Int. Dairy J..

[67] Gyawali R., Ibrahim S.A. (2016). Effects of Hydrocolloids and Processing Conditions on Acid Whey Production with Reference to Greek Yogurt. Trends Food Sci. Technol..

[68] Mehra R., Kumar H., Rafiq S., Kumar N., Buttar H.S., Leicht K., Okpala C.O.R., Korzeniowska M. (2022). Enhancing Yogurt Products’ Ingredients: Preservation Strategies, Processing Conditions, Analytical Detection Methods, and Therapeutic Delivery-An Overview. PeerJ.

[69] Aportela‚ÄêPalacios A., Sosa‚ÄêMorales M., Vélez‚ÄêRuiz J. (2005). Rheological and Physicochemical Behavior of Fortified Yogurt with Fiber and Calcium. J. Texture Stud..

[70] Mahomud M.S., Katsuno N., Nishizu T. (2017). Role of Whey Protein-Casein Complexes on Yoghurt Texture. Rev. Agric. Sci..

[71] Bierzuńska P., Cais-Sokoli≈Ñska D., Yiƒüit A. (2019). Storage Stability of Texture and Sensory Properties of Yogurt with the Addition of Polymerized Whey Proteins. Foods.

[72] Gharibzahedi S.M.T., Chronakis I.S. (2018). Crosslinking of Milk Proteins by Microbial Transglutaminase: Utilization in Functional Yogurt Products. Food Chem..

[73] Vital A.C.P., Goto P.A., Hanai L.N., Gomes-da-Costa S.M., de Abreu Filho B.A., Nakamura C.V., Matumoto-Pintro P.T. (2015). Microbiological, Functional and Rheological Properties of Low-Fat Yogurt Supplemented with Pleurotus ostreatus Aqueous Extract. LWT.

[74] Guggisberg D., Cuthbert-Steven J., Piccinali P., Bütikofer U., Eberhard P. (2009). Rheological, Microstructural and Sensory Characterization of Low-Fat and Whole Milk Set Yoghurt as Influenced by Inulin Addition. Int. Dairy J..

[75] Shaker R., Jumah R., Abu-Jdayil B. (2000). Rheological Properties of Plain Yogurt during Coagulation Process: Impact of Fat Content and Preheat Treatment of Milk. J. Food Eng..

[76] Shokery E.S., El-Ziney M.G., Yossef A.H., Mashaly R.I. (2017). Effect of Green Tea and Moringa Leave Extracts Fortification on the Physicochemical, Rheological, Sensory and Antioxidant Properties of Set-Type Yoghurt. J. Adv. Dairy Res..

[77] Najgebauer-Lejko D., Żmudziński D., Ptaszek A., Socha R. (2013). Textural Properties of Yogurts with Green Tea and Pu-erh Tea Additive. Int. J. Food Sci. Technol..

[78] de Campo C., Assis R.Q., da Silva M.M., Costa T.M.H., Paese K., Guterres S.S., de Oliveira Rios A., Flôres S.H. (2019). Incorporation of Zeaxanthin Nanoparticles in Yogurt: Influence on Physicochemical Properties, Carotenoid Stability and Sensory Analysis. Food Chem..

[79] Athar I.H., Shah M.A., Khan U.N. (2000). Effect of Various Stabilizers on Whey Separation (Syneresis) and Quality of Yogurt. Pak. J. Biol. Sci..

[80] Raftani Amiri Z., Rezaei Erami S., Jafari S.M., Ahmadian S. (2024). Physicochemical Properties of Yogurt Enriched with Nanoliposomes Containing Bitter Melon Extract. LWT.

[81] Asaduzzaman M., Mahomud M.S., Haque M.E. (2021). Heat-Induced Interaction of Milk Proteins: Impact on Yoghurt Structure. Int. J. Food Sci..

[82] Tavakoli H., Hosseini O., Jafari S.M., Katouzian I. (2018). Evaluation of Physicochemical and Antioxidant Properties of Yogurt Enriched by Olive Leaf Phenolics within Nanoliposomes. J. Agric. Food Chem..

[83] Borjizadeh Z., Ahari H., Özdal T., Khosravi-Darani K., Mohammadi Nafchi A. (2024). Saffron Nanoencapsulation (*Crocus sativus*) and Its Role in Food Science: Types and Techniques. ACS Food Sci. Technol..

[84] Radi U.M.A., Doosh K.S. (2023). Study of the Physiochemical and Sensory Properties of Therapeutic Low-Cholesterol Yoghurt Fortified with Zinc Nanoparticles. IOP Conf. Ser. Earth Environ. Sci..

[85] Schram L.B., Nielsen C.J., Porsgaard T., Nielsen N.S., Holm R., Mu H. (2007). Food Matrices Affect the Bioavailability of (n-3) Polyunsaturated Fatty Acids in a Single Meal Study in Humans. Food Res. Int..

[86] Ghorbanzade T., Jafari S.M., Akhavan S., Hadavi R. (2017). Nano-Encapsulation of Fish Oil in Nano-Liposomes and Its Application in Fortification of Yogurt. Food Chem..

[87] (2012). For the Disposal of Human Blood and Its Components for Therapeutic Purposes.

[88] (2022). General Requirements for the Competence of Testing and Calibration Laboratories.

[89] (2022). Medical Laboratories—Requirements for Quality and Competence.

[90] (2022). Clinical Laboratory Testing and In Vitro Diagnostic Test Systems.

[91] (2015). Quality Management Systems.

[92] Peña-Medina R.L., Fimbres-Olivarría D., Enríquez-Ocaña L.F., Martínez-Córdova L.R., Del-Toro-Sánchez C.L., López-Elías J.A., González-Vega R.I. (2023). Erythroprotective Potential of Phycobiliproteins Extracted from *Porphyridium cruentum*. Metabolites.

[93] Ruiz-Cruz S., González-Vega R.I., Robles-Zepeda R.E., Reyes-Díaz A., López-Elías J.A., Alvarez-Ainza M.L., Cinco-Moroyoqui F.J., Moreno-Corral R.A., Wong-Corral F.J., Borboa-Flores J. (2022). Association of Different ABO and Rh Blood Groups with the Erythroprotective Effect of Extracts from Navicula incerta and Their Anti-Inflammatory and Antiproliferative Properties. Metabolites.

[94] González-Vega R.I., Cárdenas-López J.C., López-Elías J.A., Ruiz-Cruz S., Reyes-Díaz A., Perez-Perez L.M., Cinco-Moroyoqui F.J., Robles-Zepeda R.E., Borboa-Flores J., Del-Toro-Sánchez C.L. (2021). Optimization of Growing Conditions for Pigments Production from Microalga Navicula incerta Using Response Surface Methodology and Its Antioxidant Capacity. Saudi J. Biol. Sci..

[95] AOAC (1990). Official Methods of Analysis.

[96] Benzie I.F.F., Strain J.J. (1996). The Ferric Reducing Ability of Plasma (FRAP) as a Measure of “Antioxidant Power”: The FRAP Assay. Anal. Biochem..

[97] Brand-Williams W., Cuvelier M.E., Berset C. (1995). Use of a Free Radical Method to Evaluate Antioxidant Activity. LWT.

[98] Re R., Pellegrini N., Proteggente A., Pannala A., Yang M., Rice-Evans C. (1999). Antioxidant Activity Applying an Improved ABTS Radical Cation Decolorization Assay. Free Radic. Biol. Med..

[99] Agarwal H., Shanmugam V.K. (2019). Anti-Inflammatory Activity Screening of Kalanchoe pinnata Methanol Extract and Its Validation Using a Computational Simulation Approach. Inform. Med. Unlocked.

[100] (2010). Codex Alimentarius. Codex Standard for Fermented Milks.

[101] AOCS (2007). Official Methods and Recommended Practices of the American Oil Chemists’ Society.

[102] AOAC (2000). The Official Methods of Analysis.

[103] Achanta K., Aryana K.J., Boeneke C.A. (2007). Fat-Free Plain Set Yogurts Fortified with Various Minerals. LWT.

[104] Lawless H.T., Heymann H. (2010). Sensory Evaluation of Food: Principles and Practices.

[105] Stone H., Sidel J.L. (2012). Sensory Evaluation Practices.

